# Transcription termination factor 2 (TTF2) prevents premature rRNA synthesis during mitotic exit

**DOI:** 10.26508/lsa.202603841

**Published:** 2026-10-01

**Authors:** Catarina Pedro, Laura Tovini, Catarina Peneda, Mia Maesano Krapinec, Raquel A Oliveira

**Affiliations:** 1 Católica Biomedical Research Centre (CBR), Católica Medical School, Universidade Católica Portuguesa (UCP) , Lisbon, Portugal; 2 Gulbenkian Institute for Molecular Medicine (GIMM) , Oeiras, Portugal

## Abstract

Mitosis poses major challenges to transcription, as gene expression is silenced and must be restored upon mitotic exit. These processes are regulated by Cdk1-dependent phosphorylation. Additional mechanisms, including transcription termination factor 2 (TTF2)–mediated removal of nascent transcripts, reinforce transcriptional shutdown. How these regulatory layers control different RNA polymerases and influence reactivation at mitotic exit remains poorly understood. Here, we probed how TTF2 controls transcription of distinct RNA classes, using polymerase-specific perturbations and nascent RNA labelling across mitosis. Loss of TTF2 led to accumulation of chromatin-associated transcripts during metaphase, predominantly RNA polymerase II–derived, consistent with its role in transcriptional clearance. Unexpectedly, TTF2 depletion caused premature RNA polymerase I reactivation during anaphase, resulting in unscheduled rRNA synthesis and early nucleolar protein recruitment. These findings place TTF2 as a novel regulator of RNA polymerase I reactivation at mitotic exit. Disruption of this control persists beyond mitosis, resulting in nucleolar fragmentation in interphase. These findings reveal TTF2 as an important regulator that interfaces with multiple RNA polymerases through distinct modes, coordinating transcriptional shutdown and timely reactivation across mitosis.

## Abstract (precis)

TTF2 restrains RNA Polymerase I reactivation at mitotic exit, and its loss triggers premature rRNA synthesis and nucleolar fragmentation.

## Introduction

Faithful control of gene expression programs across cell division requires not only efficient transcriptional silencing at mitotic entry but also accurate and timely transcriptional reactivation at mitotic exit. These processes are critical for divisions that maintain cellular identity as well as for those that drive changes in cell fate. Although transcriptional inactivation during mitosis has been recognised for decades (Prescott & Bender, 1962), whether and how the mechanisms that enforce transcriptional shutdown influence the subsequent reactivation of transcription remains poorly understood.

Transcriptional silencing at mitotic entry is driven primarily by the activity of the mitotic kinase Cdk1, which inhibits transcription across multiple RNA polymerase systems. For RNA polymerase I (Pol I), Cdk1/cyclin B–mediated phosphorylation of one of the SL1 complex subunits, hTAFI110, leads to its inactivation and impairs its interaction with upstream binding factor (UBF). UBF is also suggested to be inactive due to mitotic specific inhibitory phosphorylations, representing an additional layer of regulation of RNA polymerase I transcription (Klein & Grummt, 1999). In parallel to the regulation of Pol I activity, mitotic entry triggers nucleolar disassembly, in part also mediated by phosphorylation of key nucleolar components. Cdk1-mediated nucleophosmin phosphorylation abolishes its RNA binding activity and thereby modulates its localization and rRNA processing function (Okuwaki et al, 2002). Nucleolin is also known to undergo phosphorylation during mitosis, although the functional significance of this modification remains incompletely understood (Zhu et al, 1999; Dranovsky et al, 2001).

RNA polymerase II (Pol II) is similarly inhibited through Cdk1-dependent phosphorylation of the C-terminal domain (CTD) and components of the general transcription machinery, including TFIID and TFIIH (Segil, 1996; Akoulitchev & Reinberg, 1998; Enserink & Chymkowitch, 2022). Although these inhibitory modifications efficiently prevent transcription initiation, they are not sufficient to promptly silence ongoing transcription. Accordingly, additional layers of regulation act at mitotic entry to ensure full removal of nascent transcripts from chromatin. Among those, transcription termination factor 2 (TTF2), also known as Lodestar (Lds) in *Drosophila*, has been described as a key regulator of this process (Jiang et al, 2004; Carmo et al, 2023; Can et al, 2026; Tovini et al, 2026 *Preprint*). TTF2 is a helicase-like ATPase that associates with mitotic chromatin (Jiang et al, 2004; Fujisawa & Labib, 2024 *Preprint*) and in vitro can displace elongating RNA polymerases from template DNA (Xie & Price, 1997; Liu et al, 1998; Hara et al, 1999). Consistently, depletion of TTF2/Lds leads to retention of elongating Pol II and nascent mRNAs on mitotic chromosomes in *Drosophila* embryos and mammalian cells. Although biochemical studies have suggested that TTF2 can also act on stalled Pol I complexes, its role in regulating rRNA transcription in vivo has not been established. In parallel to TTF2 activity, other players have also been proposed to aid mitotic transcriptional silencing, including aurora B–dependent phosphorylation of SAF-A, as well as removal of cohesin from chromosome arms, which triggers the eviction of nascent RNAs and Pol II from chromatin (Perea-Resa et al, 2020; Sharp et al, 2020).

Together, these studies define a multilayered network that ensures transcription is efficiently switched off at mitotic entry. By contrast, how transcription is subsequently reactivated remains far less clear. Reactivation could be viewed simply as the reversal of inhibitory phosphorylations and the reassembly of transcription complexes. However, given the extent of chromatin reorganization during mitosis, it is conceivable that transcriptional reactivation follows a regulated and ordered sequence of events, rather than occurring uniformly across transcriptional systems. Accordingly, present models propose that Pol I is reactivated in a multistep manner. Upon mitotic exit, hTAFI110 is dephosphorylated and SL1 rapidly reactivated, driving the initial stages of rRNA transcription in telophase (Heix, 1998; Fu et al, 2023). However, this was shown insufficient for full restoration of maximal Pol I activity, which was proposed to occur later in G1, upon UBF’s activating phosphorylation by G1-specific Cdks/cyclins (Voit, 1999; Voit & Grummt, 2001). Furthermore, the TATA-binding protein (TBP) has also been shown to bookmark rDNA loci during mitosis, a binding event which is necessary for proper and timely reactivation of Pol I transcription after mitosis (Kwan et al, 2024).

In summary, despite a growing understanding of how Pol II–dependent transcription is silenced during mitosis, how these silencing mechanisms act upon other RNA polymerases, and whether or not they shape the timing, order, and coordination of transcriptional reactivation remains poorly understood. In this study, we investigate TTF2-mediated transcriptional clearance towards different RNA types and uncovered an unanticipated role for TTF2 in restraining premature Pol I reactivation at mitotic exit. Our findings reveal a new layer of regulation that mechanistically links transcriptional inactivation with ordered transcriptional reawakening.

## Results

### TTF2 leads to the accumulation of morphologically different types of nascent RNA across mitotic stages

**Figure S1.**
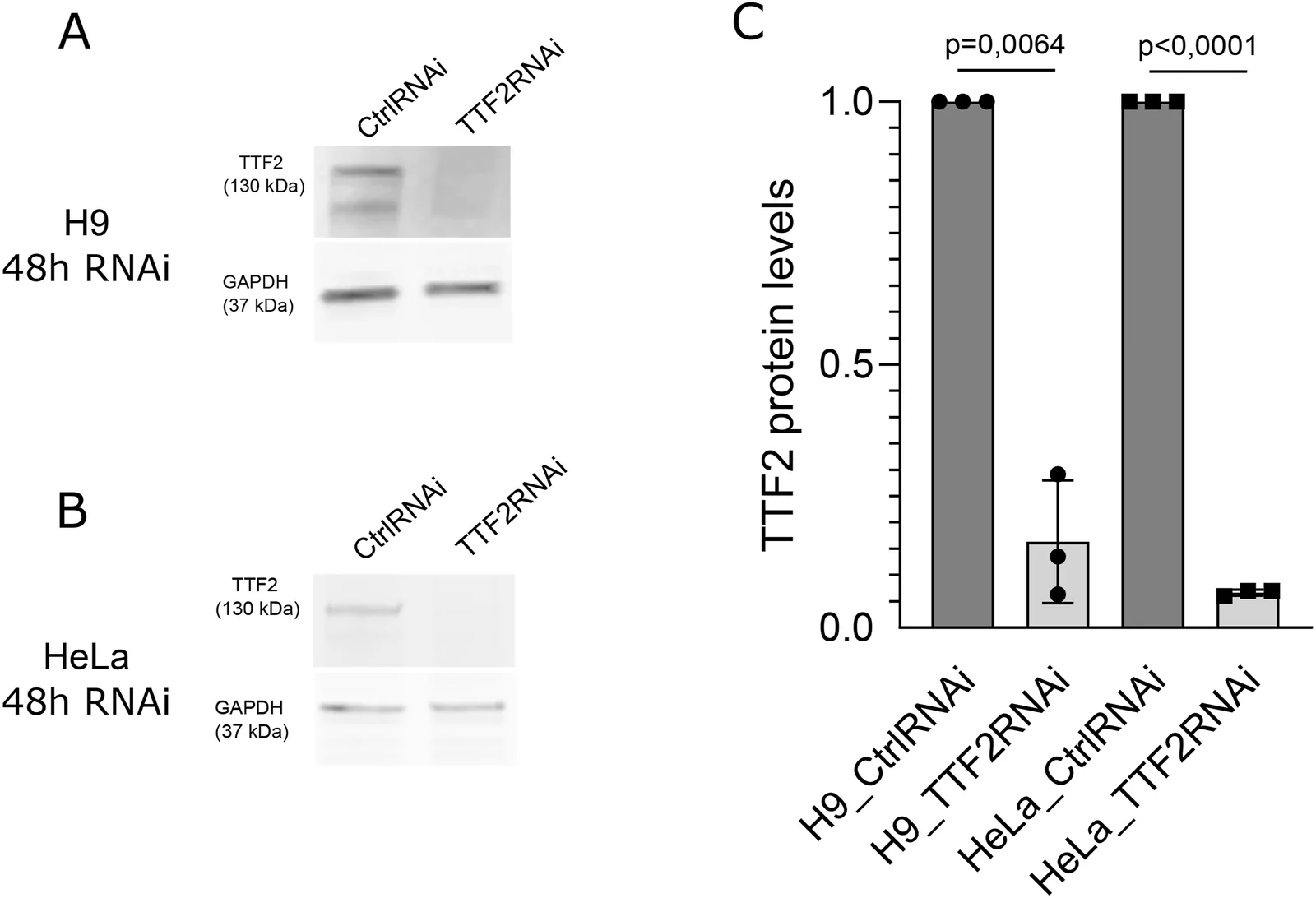
Depletion of TTF2 in H9 human embryonic stem cells and HeLa cancer cells. **(A, B)** Western blot of Control RNAi and TTF2RNAi conditions in H9 (A) and HeLa cells (B) at 48 h of RNAi treatment. **(C)** Quantification of TTF2 protein depletion levels in H9 and HeLa cells. Data are derived from three independent experiments for each cell line. Error bars represent mean ± SD. Statistical analysis on TTF2 knockdown efficiency in TTF2RNAi compared with CtrlRNAi, was performed using two-tailed paired *t* test for both H9 and HeLa cells. *P* < 0.05 is considered statistically significant.

**Figure 1.**
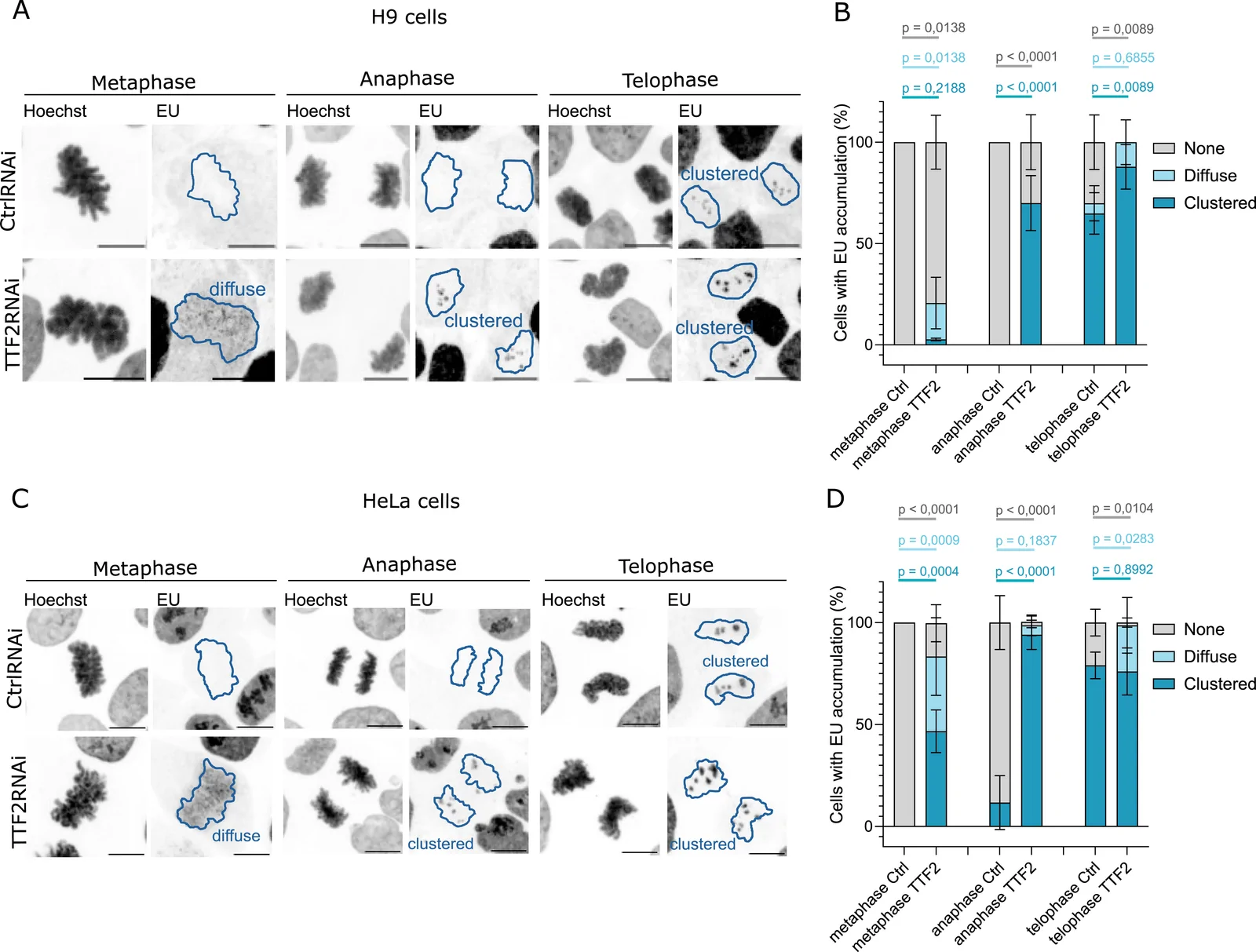
TTF2 knockdown leads to accumulation of nascent RNA transcripts across mitosis in both H9 human embryonic stem cells and HeLa cancer cells. **(A, C)** Representative images of H9 (A) and HeLa cells (C) in Control and TTF2RNAi conditions with nascent RNA labelling (EU), across the different stages of mitosis. **(B, D)** Percentage of cells with none, diffuse, or clustered EU signal across the different stages of mitosis indicated. Data from three independent experiments, error bars indicate the mean ± SD. Statistical analysis was performed using Firth’s penalized logistic regression, comparing TTF2RNAi to CtrlRNAi for each category (“none,” “diffuse,” and “clustered”). *P*-values were corrected for multiple comparisons using the Benjamini–Hochberg false discovery rate (FDR) method. *P* < 0.05 is considered statistically significant. Scale bars are 10 μm.

To investigate the role of TTF2 on transcription dynamics across mitotic stages, we induced TTF2 depletion in two distinct cell lines, H9 human embryonic stem cells and HeLa cells (Fig S1A–C), by transfecting control or TTF2-specific siRNAs for 48 h. At this timepoint, a 30-min 5′ ethynil uridine (EU) pulse was performed to label nascent RNA. EU incorporation was then detected using Click-iT chemistry, and chromosomes were counterstained with Hoechst, enabling simultaneous visualization of mitotic chromosomes and newly synthesized RNA (Fig 1).

In both cell lines, we observed that in Control RNAi, EU-labelled transcription is virtually absent in metaphase/anaphase cells. In contrast, upon TTF2 depletion, nascent RNAs are present throughout the entire stages of mitosis (Fig 1). The phenotype was more penetrant in HeLa cells, compared with H9, particularly when considering the total frequency of metaphase cells with EU (82% in HeLa versus 21% H9, Fig 1D and B). Detailed analysis of the distribution of observed EU signals allowed us to distinguish two distinct phenotypes: a more *diffuse* pattern, characterised by a dispersed signal covering most of the chromatin, and a *clustered* pattern, in which strong signals are confined to discrete sub-chromosomal regions, with little to no signal detected across the remaining chromatin (Fig 1A and C). In both cell types, upon TTF2 depletion, a significant proportion of metaphases exhibit diffuse signals (17% in H9 and 36% in HeLa cells, Fig 1B and D). Clustered signals appear in 46% of metaphases in HeLa cells but are nearly absent in H9 (4%) (Fig 1B and D). Strikingly, anaphase cells show a high percentage of clustered signals in both cell types (69% in H9 and 94% in HeLa).

The presence of a diffuse signal all over metaphase chromatin is compatible with the role of TTF2 in the eviction of Pol II and nascent mRNAs (Jiang et al, 2004; Can et al, 2026; Tovini et al, 2026 *Preprint*). However, defects in clearance should lead to a constant or decreased EU signal across mitotic progression. Therefore, the high frequency and clustered appearance of EU signals in anaphase is less clear to interpret and suggests a different activity of TTF2 towards specific chromosomal regions. This was particularly striking in H9 cells, where the frequency of anaphase clusters was much higher than the total of metaphases with any detectable EU signal on chromatin (69% versus 20%, Fig 1B). We therefore focused on exploring the nature of the clustered EU signals during anaphase, using H9 as the prime model system.

### TTF2 depletion triggers an earlier reactivation of ribosomal RNA transcription during mitotic exit

Given the higher frequency of the signals observed during anaphase upon TTF2 depletion, and their clustered appearance, we hypothesised that this signal might correspond to rRNA transcripts because these are transcribed from repetitive rDNA regions that are clustered in the genome (Sultanov & Hochwagen, 2025).

**Figure S2.**
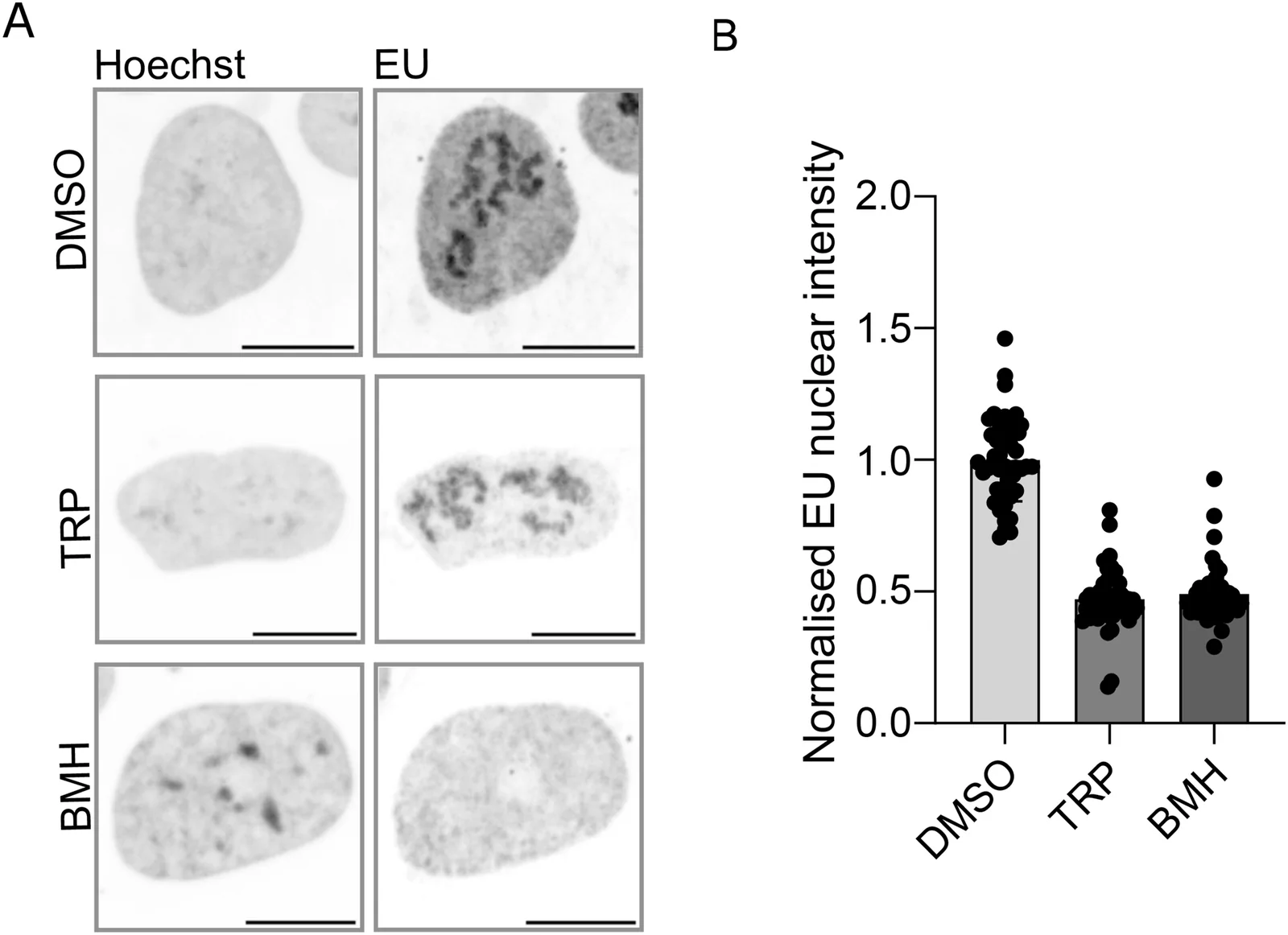
TRP and BMH efficiently inhibit RNA polymerase II and RNA polymerase I activity in H9 cells. **(A)** Representative images of interphase H9 cells in control DMSO, TRP, or BMH (2 h) treatment, showing the nascent RNA labelling (EU) and its decrease on the nucleoplasm and on the nucleolus upon TRP and BMH treatment, respectively. **(B)** Nuclear EU signal intensity normalised to the mean of the control (DMSO). Each dot represents one cell, data from one experiment. Scale bars are 10 μm.

We therefore combined differential inhibition of specific RNA polymerases (triptolide (TRP) for Pol II and BMH-21 for Pol I [Titov et al, 2011; Ferreira et al, 2020]) with TTF2 depletion. A 2-h TRP or BMH-21 treatment was included at the end of the previously described siRNA transfection and EU incorporation protocol, and it was sufficient to decrease the amount of nascent RNAs (EU signal) detected in the nucleoplasm or nucleolar regions, respectively, of interphase cells (Fig S2A and B), confirming the drugs’ efficacy.

**Figure 2.**
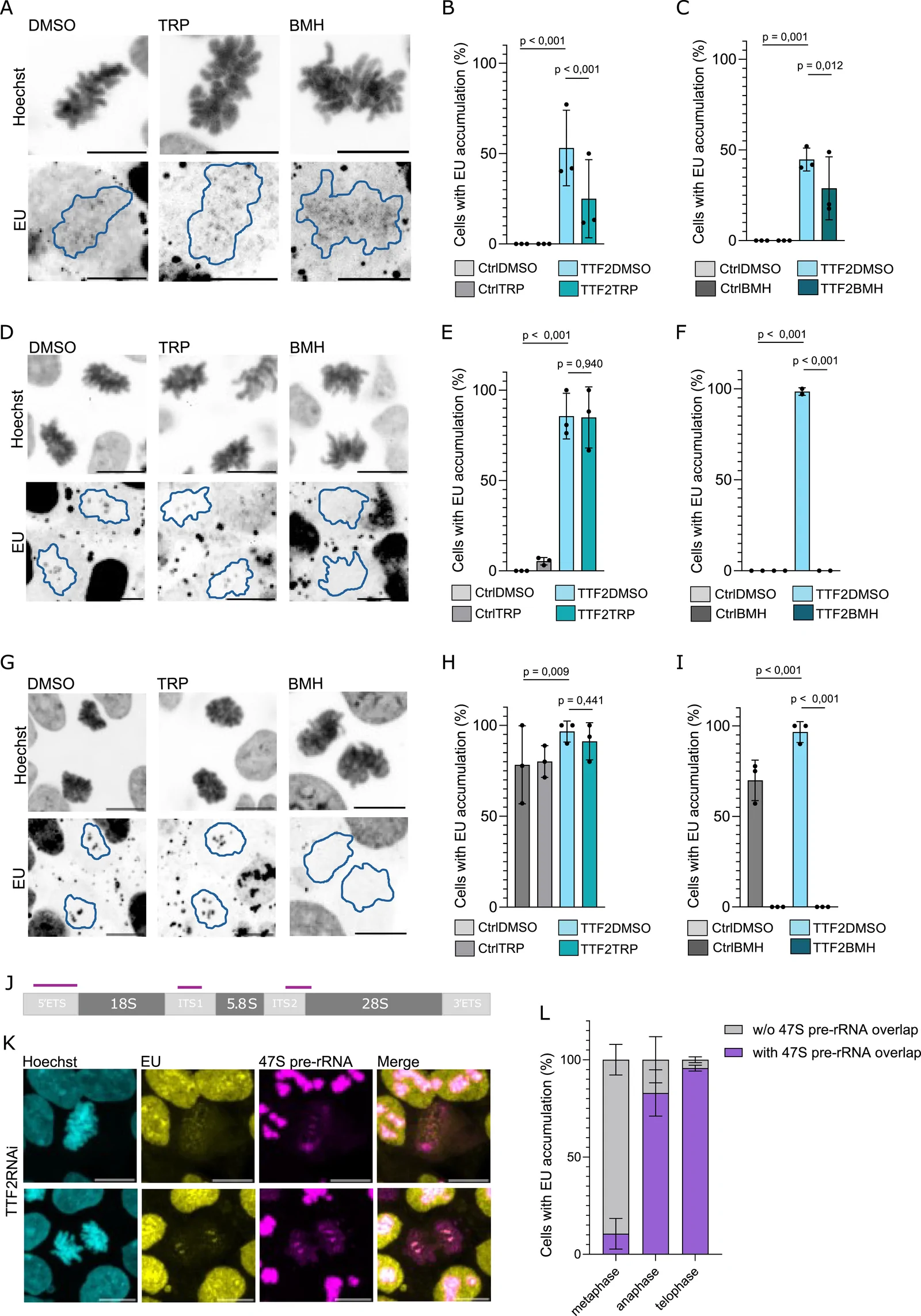
Nascent RNA transcripts in H9 anaphase cells depleted for TTF2 are a consequence of an earlier reactivation of ribosomal RNA transcription. **(A, D, G)** Representative images of nascent RNA labelling in metaphase (A), anaphase (D) and telophase (G) H9 cells in Control and TTF2RNAi conditions upon DMSO, TRP, or BMH treatments. **(B, C, E, F, H, I)** Percentage of cells displaying nascent RNA labelling (EU). Data from three independent experiments; error bars indicate mean ± SD. For each mitotic stage (metaphase, anaphase, telophase), the percentage of cells with or without signal was compared pairwise (CtrlRNAiDMSO versus TTF2RNAiDMSO; TTF2RNAiDMSO versus TTF2RNAiTRP; TTF2RNAiDMSO versus. TTF2RNAiBMH) using Firth’s penalized logistic regression with replicate included as a covariate. Resulting *P*-values were corrected for multiple comparisons using the Benjamini–Hochberg procedure and are represented. FDR-adjusted *P* < 0.05 was considered statistically significant. **(J)** Schematics of the 47S pre-rRNA gene unit with the sites where the RNA FISH probes bind to (purple line). **(K)** Representative images of TTF2-depleted cells, with or without (w/o) overlap between EU and 47S pre-rRNA FISH probes. **(L)** Percentage of cells with nascent RNA labelling with or without overlap between EU and 47S rRNA signal. Two independent experiments analysed, error bars indicate the mean ± SD. Scale bars are 10 μm.

We then assessed the consequences of each inhibitor in TTF2-depleted metaphase cells. TRP treatment significantly decreased the amount of metaphase cells that display EU accumulation on the chromatin by around 50% (Fig 2A and B). On the opposite, BMH-21 had a similar effect, albeit to a lower extent (∼37% reduction) (Fig 2A and C). These findings suggest that nascent transcripts on metaphase chromatin may result from a mixture of mRNA and rRNA transcription events.

A similar analysis in TTF2-depleted anaphase and telophase cells produced a much-differentiated response. Whereas TRP treatment had no impact on the frequency of signals detected at these late mitotic stages (Fig 2D, E, G, and H), BMH-21 fully abolished the presence of nascent transcripts on anaphase and telophase chromatin (Fig 2D, F, G, and I). These results indicate that clustered signals result from Pol I–mediated rRNA transcription.

**Figure S3.**
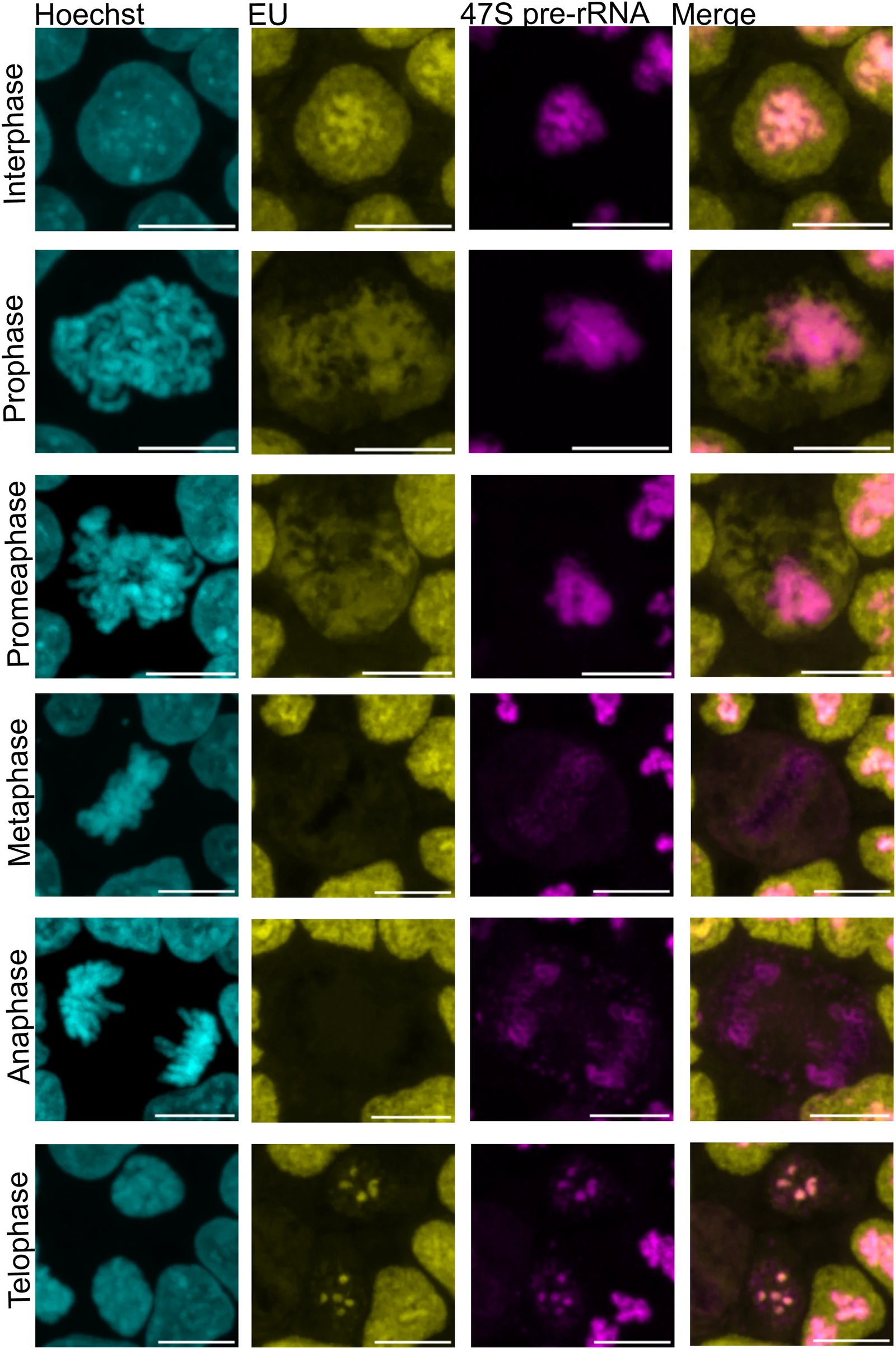
47S pre-rRNA FISH probes for detection of the ribosomal RNA primary transcript. Representative images of H9 cells of interphase cells and across the mitotic stages indicated. Scale bar is 10 μm.

To confirm this possibility, we combined the Click-iT chemistry protocol for nascent RNA labeling with RNA FISH probes designed to target the 47S pre-rRNA (Fig 2J). In control interphase cells, this probe shows an overlap with high intensity EU-labelled nascent RNA (inferred as the nucleolus, Fig S3), confirming its specificity.

We next focused on the analysis of mitotic cells. In TTF2-depleted metaphase cells, we observed very little overlap between nascent RNA (EU signal) and the RNA FISH probes (∼10% of EU-positive metaphase cells). In contrast, in anaphase cells, this co-localisation occurred in 80% of the analysed cells (Fig 2K and L). These results indicate that upon TTF2 depletion, the RNAs accumulated on metaphase chromatin are poor in Pol I transcripts. In sharp contrast, in anaphase, only Pol I–derived transcripts are detected. Considering the high frequency of nascent rRNAs observed in anaphase cells, despite their nearly absence in metaphase cells (Fig 2L), we infer that rRNAs detected in anaphase/telophase result from a premature transcriptional reactivation rather than a perdurance of G2 transcripts.

### TTF2 depletion leads to premature transcription of the entire rRNA unit during mitotic exit

So far, our results suggest that TTF2 depletion induces an earlier reactivation of rDNA transcription. However, it is unclear whether this anticipated early onset results in processive elongation across the full transcriptional unit or allows initiation without sustained elongation, leading to transcriptional stalling.

**Figure 3.**
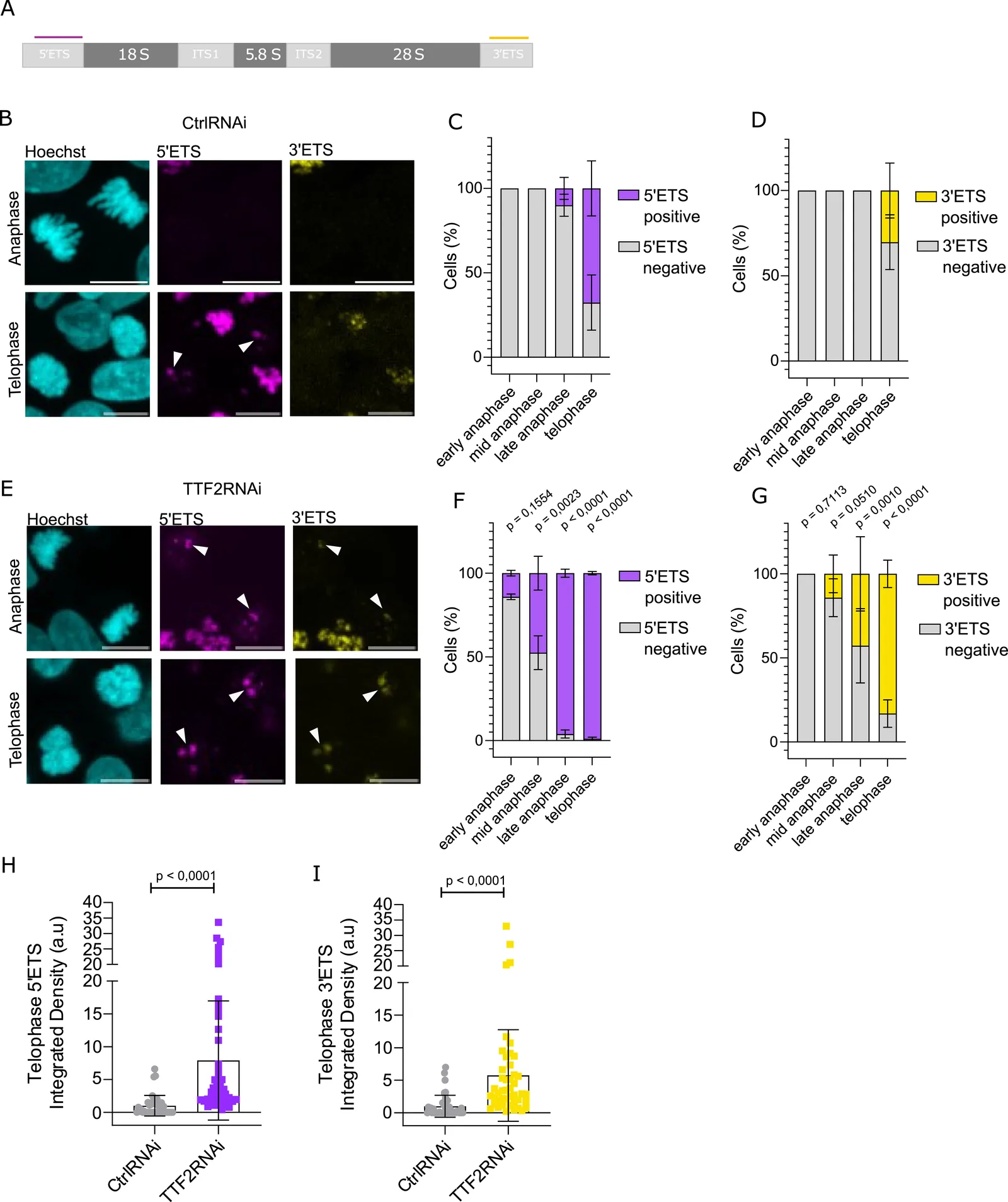
TTF2 depletion in H9 embryonic stem cells leads to an earlier and fully productive reactivation of rDNA transcription. **(A)** Schematics of the 47S pre-rRNA gene unit and the sites where the 5′ETS (purple) and 3′ ETS (yellow) RNA FISH probes bind to. **(B, E)** Representative images of anaphase and telophase H9 cells in Control and TTF2RNAi conditions, displaying the 5′ETS and 3′ETS. **(C, D, F, G)** Percentage of cells in Control and TTF2-depleted cells that display the 5′ETS signal (C, F), and 3′ETS signal (D, G). Error bars indicate the mean ± SEM from three independent experiments. The proportion of cells positive for 5′ETS signal or 3′ETS signal in TTF2RNAi (F, G) was compared with the respective cells in CtrlRNAi (C, D) for each mitotic stage (“early anaphase,” “mid anaphase,” “late anaphase,” and “telophase”), using Firth’s penalized logistic regression. Respective *P*-values displayed in (F) and (G) represent this comparison. *P* < 0.05 is considered statistically significant. Quantifications of the 5′ETS (H) and 3′ETS (I) signal in Control and TTF2RNAi telophase cells, where each dot represents one cell. Error bars indicate the mean ± SD of the analysed cells, derived from three independent experiments. Statistical analysis was performed using the nonparametric Mann–Whitney test. Scale bars are 10 μm.

To distinguish between these two possibilities, we designed RNA FISH probes specifically targeting the 5′ external transcribed spacer (5′ETS), which marks the 5′ end of the 47S pre-rRNA transcript or the 3′ ETS, corresponding to the 3′ terminus of the transcript (Fig 3A). To gain further temporal resolution, we focused on anaphase and telophase cells and categorized them as early-, mid-, or late-anaphase (based on the distance between segregating chromatids) and telophase (when chromatin acquired a round shape). In control conditions, the 5′ETS RNA probe is absent in early/mid anaphases and only visible in some late anaphases (14%), and more consistently in telophase cells (84%) (Fig 3B and C). In turn, the 3′ETS signal, indicating transcription of the end of gene, is only visible in about half of telophase cells (Fig 3B and D).

When evaluating the time and localization of these same probes in TTF2-depleted cells, we detect expression of the 5′ETS region earlier than in control cells, including some early anaphases (∼12%) (Fig 3E and F). In agreement with the dynamics of a productively elongating Pol I, the region of the 3′ETS corresponding to the end of the gene can be observed already in mid-anaphase cells (20%) (Fig 3E and G). Based on these observations, we conclude that not only Pol I starts transcribing earlier upon TTF2 depletion but also that it can transcribe the entire rDNA gene before cells reach telophase. To estimate the amount of nascent transcripts, in Control and TTF2RNAi conditions, we quantified the signal from each probe. This analysis revealed that in telophase, TTF2-depleted cells display higher levels of both the 5′ETS and 3′ETS probes (Fig 3H and I). We therefore conclude that prematurely reactivated Pol I can transcribe the entire transcriptional unit, leading to higher amounts of full 47S pre-rRNA transcripts produced in telophase cells.

### Premature rRNA transcription is sufficient to trigger partial nucleolar reassembly

Given the known interplay between rDNA transcription and nucleolar assembly (Falahati et al, 2016; Dash et al, 2023), we next sought to evaluate whether premature rRNA transcription, observed upon TTF2 depletion, was sufficient to initiate nucleolar reassembly.

**Figure 4.**
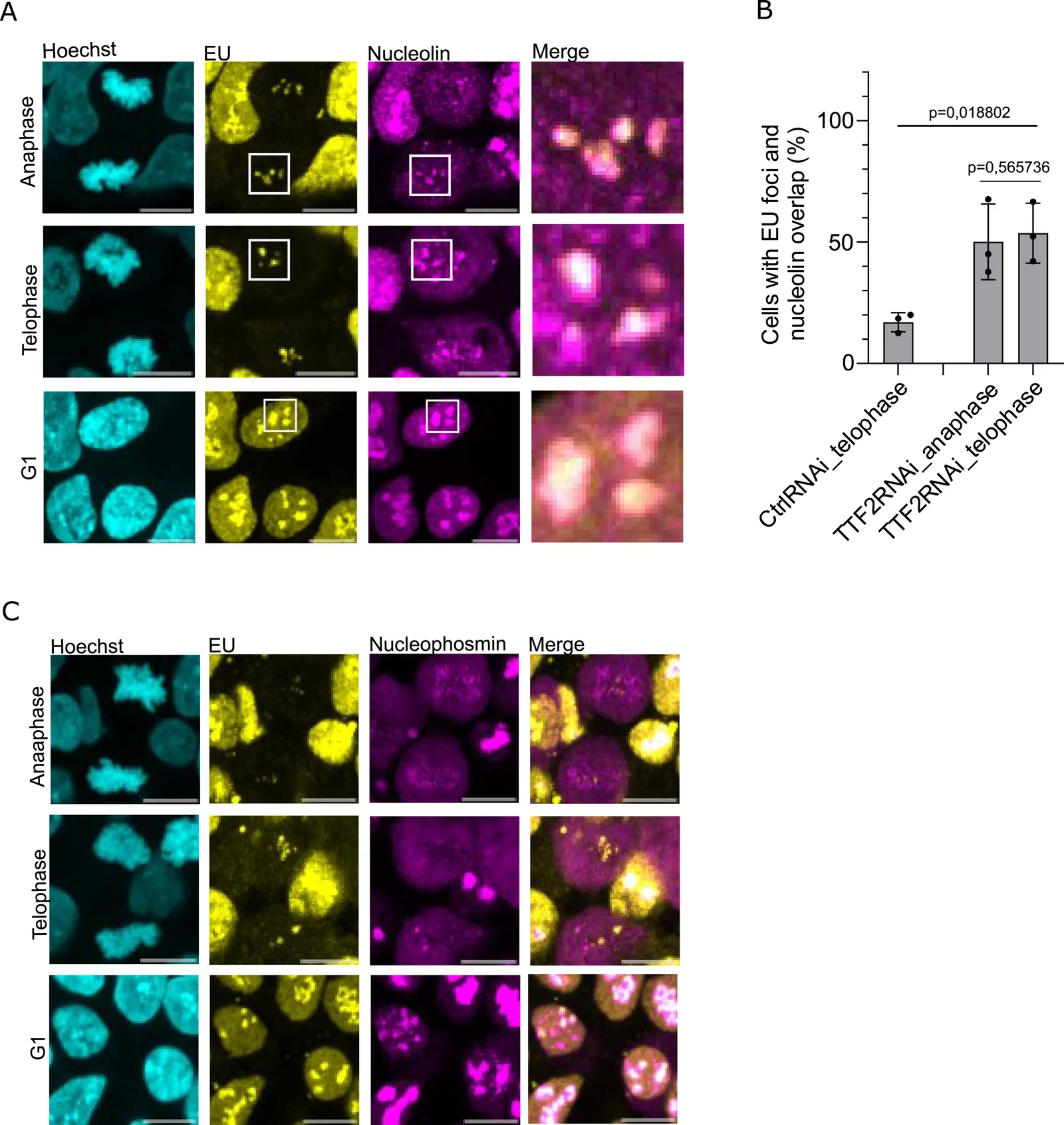
Anticipated rRNA transcription induced by TTF2 depletion is concomitant with earlier recruitment of nucleolin, but not nucleophosmin. Representative images of cells with nascent RNA labelling and stained with nucleolin (A) and nucleophosmin (C) in the anaphase, telophase, and G1 TTF2-depleted cells. **(B)** Percentage of cells that show an overlap between EU and nucleolin, in control telophase cells and TTF2-depleted anaphase and telophase cells. Error bars indicate the mean ± SD from three independent experiments. Statistical analysis was performed on cell count, using Fisher’s exact test. *P* < 0.05 is considered statistically significant. Scale bars are 10 μm.

We used two commonly used nucleolar markers, nucleolin and nucleophosmin, to evaluate their co-localisation across the entire EU-labelled region, using only EU-positive cells for this analysis (telophase in controls and anaphase/telophase in TTF2) (Fig 4).

**Figure S4.**
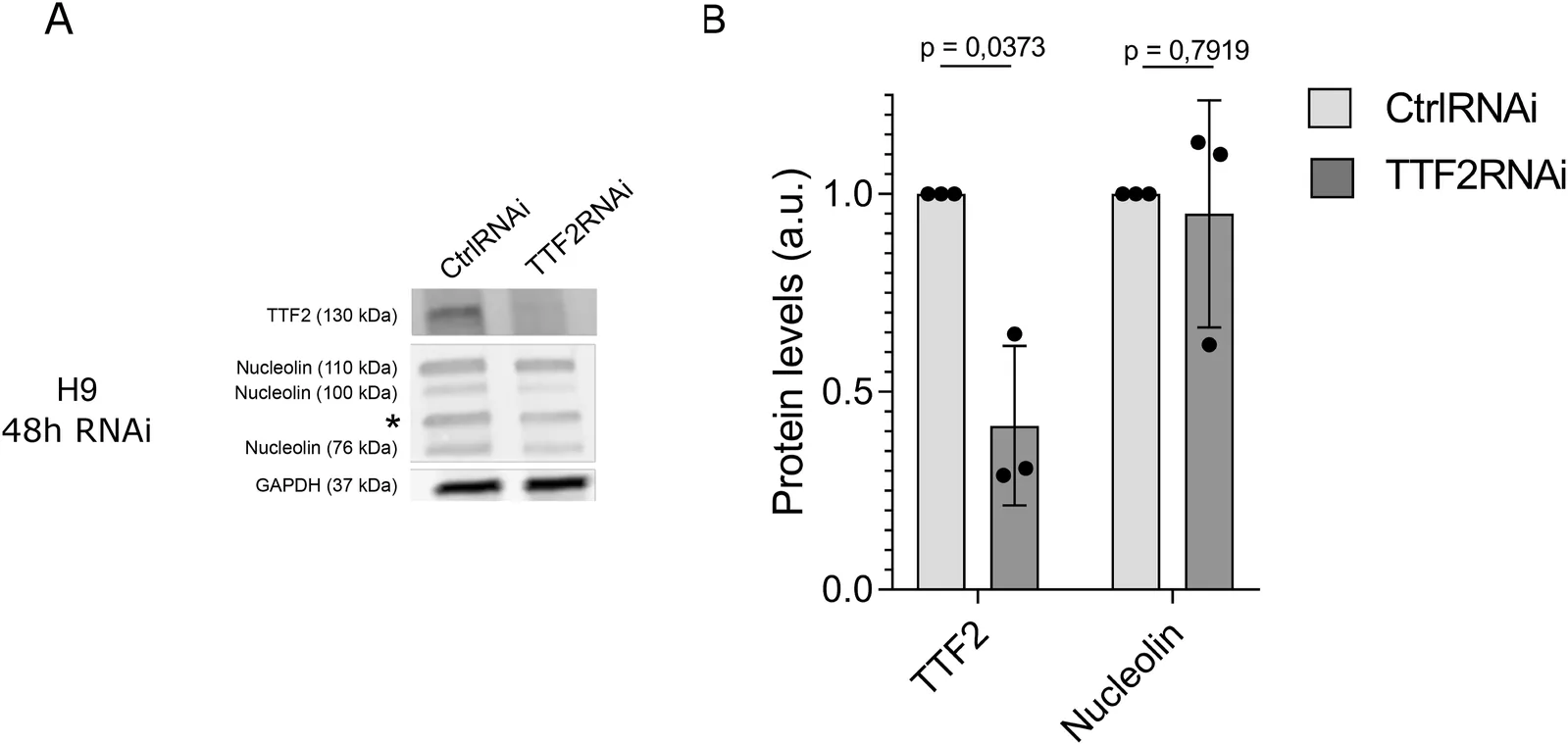
Nucleolin protein levels remain unaltered upon TTF2 depletion in H9 human embryonic stem cells. **(A)** Western blot for TTF2 and nucleolin (110-, 100-, and 76-kD forms) in H9 CtrlRNAi and TTF2RNAi conditions at 48 h of RNAi treatment, with GAPDH as loading control, and (B) respective quantifications. Nucleolin levels were quantified by summing the raw values of each identified nucleolin band and then normalized to the respective loading control. Unspecified nucleolin band is identified by *. Data are from three independent experiments. Error bars indicate mean ± SD. Statistical analysis on TTF2 and nucleolin protein levels in TTF2RNAi compared with CtrlRNAi, was performed using two-tailed paired *t* test. *P* < 0.05 is considered statistically significant.

For nucleolin, we observed that in control cells, a full overlap with EU-labelled foci was detected in 17% of telophases. These findings suggest that the recruitment of nucleolar components occurs after the onset of rRNA transcription. For TTF2-depleted cells, these percentages are higher in both anaphase and telophase (50% and 53%, respectively) (Fig 4A and B). These findings suggest that anticipated rDNA transcription is sufficient to trigger nucleolin recruitment to rRNA transcription sites as early as anaphase. Notably, these observations are not attributable to increased protein levels, as the most commonly described forms of nucleolin protein (76, 100, and 110 kD) (Bicknell et al, 2005) remain unchanged after TTF2 depletion (Fig S4A and B).

In contrast, when assessing the localization of nucleophosmin, known to be recruited much later in G1 (Leung et al, 2004; Fu et al, 2023), we observed no co-localization between EU-labelled regions in anaphase and telophase (Fig 4C). These findings suggest that premature transcription of rRNA can initiate the recruitment of some nucleolar components, but not all.

### TTF2 depletion impairs nucleolar integrity in interphase cells

Our findings show that rRNA production, upon TTF2 depletion, is prematurely activated at mitotic exit, with concomitant partial nucleolar assembly. As such, we sought to determine whether this condition affects nucleolar structure beyond mitotic stages.

**Figure 5.**
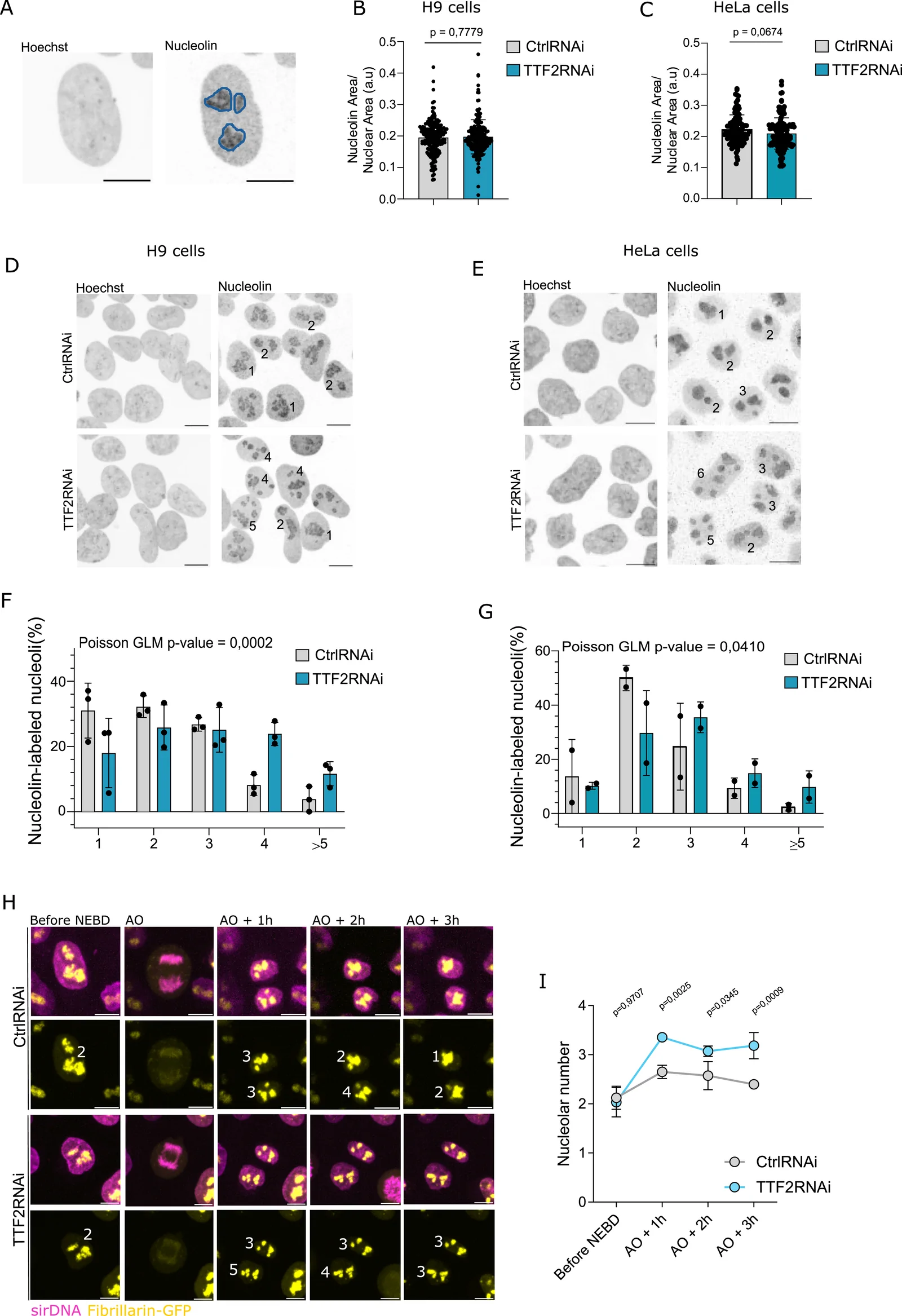
TTF2 depletion in H9 embryonic stem cells and HeLa cancer cells causes an increase in nucleoli numbers. **(A)** Nucleolin staining was used to define nucleolar area and count nucleoli numbers. **(B, C)** Nucleolin area normalised by nuclear area for H9 cells and HeLa cells. Each dot represents one cell, error bars indicate the mean ± SD. Statistical significance was determined by two-tailed Mann–Whitney U test. *P* < 0.05 is considered statistically significant. **(D, E)** Representative images of H9 (D) and HeLa (E) interphase cells with the indicated number of nucleolin-labelled nucleoli in Control and TTF2RNAi conditions. **(F, G)** Percentage of control or TTF2-depleted cells (H9 and HeLa cells, F, G) displaying the indicated number of nucleoli. Three independent experiments analysed for H9 cells and two independent experiments for HeLa. Statistical analysis was performed on cell count, using Poisson generalized linear model (GLM). Error bars indicate the mean ± SD. Scale bars are 10 μm. **(H)** Representative live-cell images of Hela cells with endogenously GFP-tagged fibrillarin, in CtrlRNAi and TTF2RNAi conditions, across different stages of the cell cycle (“before NEBD,” “AO,” “AO+1 h,” “AO+2 h,” and “AO+3 h”) and (I) respective nucleoli number quantifications. Data are from three independent experiments. Error bars indicate the mean ± SD. Statistical analysis was assessed by two-way ANOVA comparing CtrlRNAi versus TTF2RNAi at each time point.

**Figure S5.**
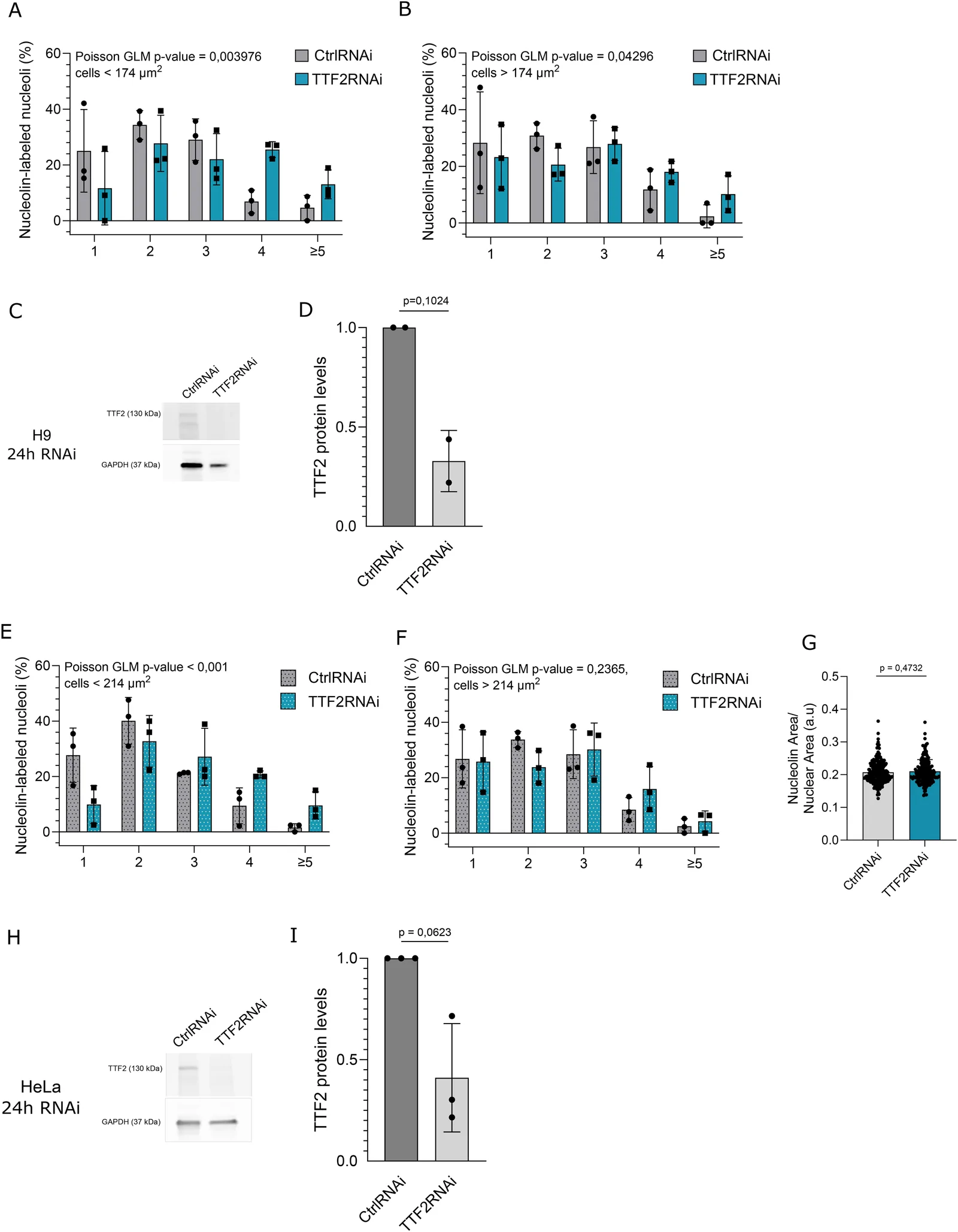
Increased nucleolar number in H9 cells is more pronounced in smaller cells (representing G1/early S-phase). **(A, B)** Nucleolar distribution in control or TTF2-depleted H9 cells at 48 h of RNAi treatment, for (A) smaller cells (likely representing G1/early S-phase) and (B) bigger cells (representing late S-phase/G2). Data are from the same three independent experiments displayed in Fig 5F. Statistical analysis was performed on cell count, using Poisson generalized linear model (GLM). Error bars indicate the mean ± SD. **(C, D)** Western blot representing TTF2 knockdown efficiency in H9 cells at 24 h of RNAi treatment (C) and respective quantifications of TTF2 protein levels in CtrlRNAi and TTF2RNAi (D). Protein extracts were derived from two independent experiments. Error bars represent mean ± SD. Statistical analysis on TTF2 knockdown efficiency in TTF2RNAi compared with CtrlRNAi at 24 h, was performed using two-tailed paired *t* test. **(E, F, G)** Nucleolar distribution was analysed at 24 h of RNAi treatment in H9 cells for Control and TTF2RNAi again for (E) smaller cells (likely representing G1/early S-phase) and (F) bigger cells (representing late S-phase/G2). Three independent experiments were analysed and statistical analysis was performed on cell count, using Poisson generalized linear model (GLM). Error bars indicate the mean ± SD. The same data were used to determine nucleolin area normalised by nuclear area (G). Each dot represents one cell, error bars indicate the mean ± SD. Statistical significance was determined by two-tailed Mann–Whitney U test. **(H, I)** Western blot representing TTF2 knockdown efficiency in HeLa cells at 24 h of RNAi treatment (H) and respective quantifications of TTF2 protein levels in CtrlRNAi and TTF2RNAi (I). Protein extracts were derived from three independent experiments. Error bars represent mean ± SD. Statistical analysis on TTF2 knockdown efficiency in TTF2RNAi compared with CtrlRNAi at 24 h, was performed using two-tailed paired *t* test.

We first measured the nucleolar area (normalised to the respective nuclear size) of interphase cells, using nucleolin as a marker (Fig 5A). This analysis revealed no significant differences in nucleolar area between Control and TTF2RNAi (Fig 5B). However, when determining the number of nucleoli per cell, we observed that TTF2-depleted H9 cells displayed a higher count of individual nucleoli (Fig 5D and F). Whereas most control cells have 1–2 nucleolar bodies upon TTF2 depletion, a high percentage of cells (∼ 60%) display 3 or more nucleoli, indicative of nucleolar fragmentation and/or nucleolar fusion defects. To confirm that increased nucleolar number is a consequence of mistimed rRNA reactivation at mitotic exit, we repeated this analysis in TTF2 siRNA–treated H9 cells 24 h after transfection (70% depletion of TTF2 protein levels, Fig S5C and D). We reasoned that in this experimental condition, G1/early S-phase cells would represent cells that have undergone mitosis in the absence of TTF2. In contrast, late S/G2 cells are enriched in cells which have undergone their last mitosis with sufficient TTF2 levels. For this distinction, we used nuclear size as a proxy for cell cycle stage. To better analyse these distributions, we divided 24-h treated cells according to the nuclear size below or above the mean population size (214 ± SD, n = 510 cells). Although the nucleolar area normalised to the respective nuclear size remained unaltered (Fig S5G), we observed that smaller TTF2-depleted cells (likely representing G1/early S-phase) have a nucleolar distribution significantly different from their respective controls (Fig S5E). In contrast, larger cells do not show significant differences (Fig S5F). 48 h after TTF2 depletion, both small and large cells show a significantly different distribution, as expected for a less temporally resolved experimental condition (Fig S5A and B).

To probe whether this phenotype was consistent across different cell types, we performed an identical analysis in HeLa cells. Similarly, no significant differences in nucleolar area between Control and TTF2RNAi were observed (Fig 5C). When comparing the number of nucleoli displayed by interphase cells, 48-h TTF2–depleted HeLa cells also showed an increased number of nucleoli. Whereas control cells mainly display two nucleoli, 54% of TTF2-depleted cells have three or more nucleoli (Fig 5E and G), also pointing to nucleolar architectural defects in these cells.

To test if nucleolar number increase is a consequence of mitotic-related defects, we used live cell imaging in HeLa cells expressing GFP-tagged fibrillarin, an important nucleolar component (Schichler et al, 2026) to evaluate nucleolar number in G2 and G1 stages. A 24-h TTF2 depletion was performed to reduce cumulative effects (Fig S5H and I).

This analysis reveals that TTF2-depleted G2 cells do not show a statistically different nucleolar number, consistent with the notion that in their previous mitosis, TTF2 levels were likely unperturbed. In sharp contrast, early G1 cells (1, 2, and 3 h post anaphase onset), show significantly higher nucleolar numbers after TTF2 depletion (Fig 5H and I).

Thus, in the two cell types analysed, TTF2 depletion is linked to abnormal nucleolar organization during interphase, likely as a consequence of their mistimed reactivation during mitotic exit.

## Discussion

In this study, we uncovered a previously unrecognised regulation of Pol I reactivation and how it uncouples rRNA transcription from cell cycle progression.

**Figure S6.**
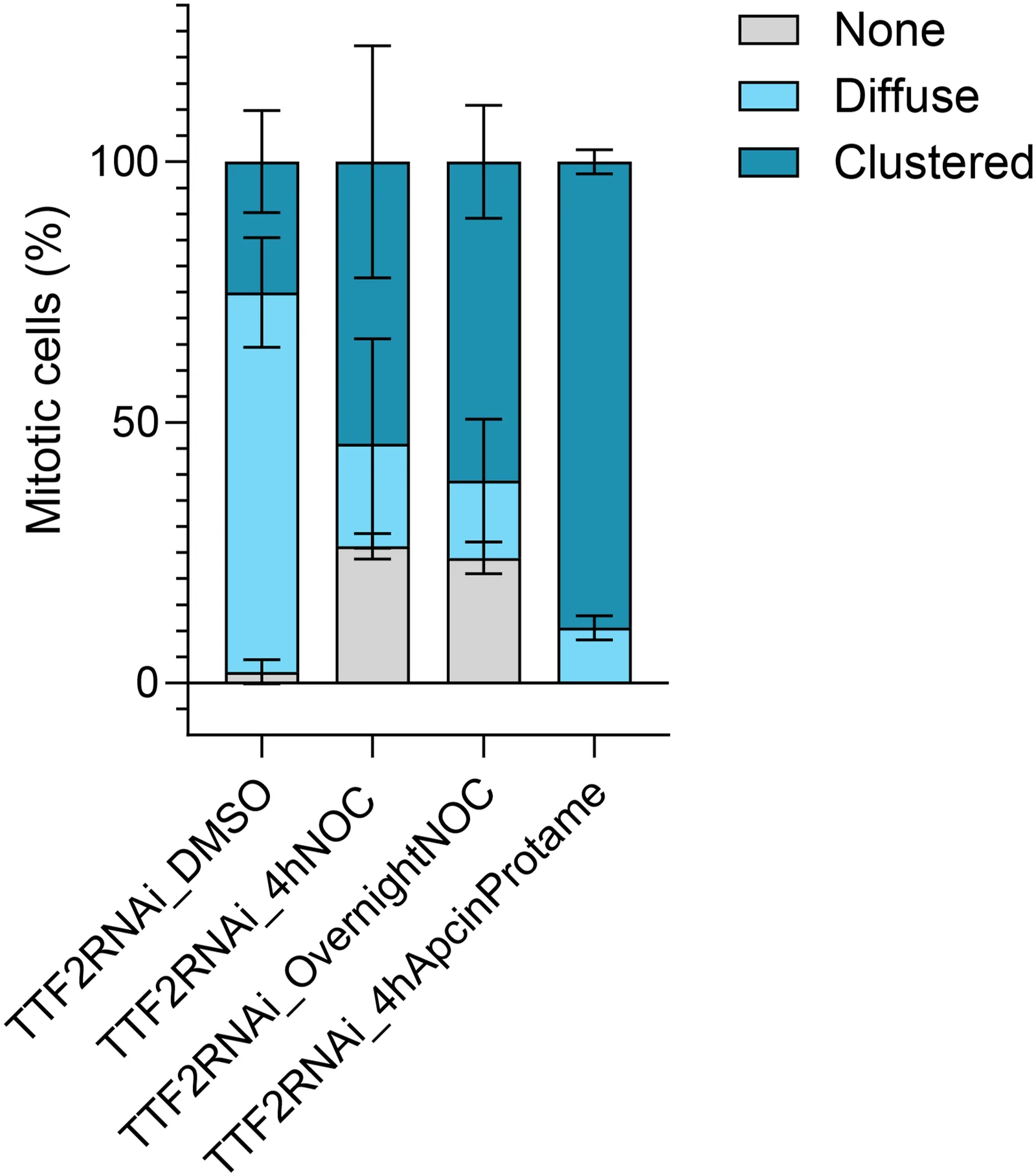
Prolonged mitotic arrest in TTF2-depleted HeLa cells results in the loss of diffuse EU signal, whereas clustered RNA polymerase I–derived EU signal persists. Percentage of TTF2-depleted, mitotically arrested HeLa cells, with either nocodazole (NOC) (4-h or overnight incubation) or Apcin–proTAME (4-h incubation), displaying no EU signal, diffuse EU signal, or clustered EU signal. Data are from three independent experiments; error bars represent mean ± SD.

Whereas the role of TTF2 in the clearance of Pol II–dependent transcripts has been previously established (Jiang et al, 2004; Can et al, 2026; Tovini et al, 2026 *Preprint*), our data reveal a novel role for TTF2 in the regulation of Pol I activity across mitosis, particularly during its reactivation at mitotic exit. We observed evidence for Pol I regulation in both cell types analysed (H9 and HeLa cells), suggesting a general role for TTF2 in this process. Evidence for premature reactivation as cells exit mitosis is particularly clear in H9 cells, where Pol I–associated transcripts are ultimately cleared even in the absence of TTF2 (by metaphase), yet reappear prematurely on chromatin during late mitotic stages. In contrast, rRNA transcripts persist on chromatin throughout mitosis in HeLa cells, making it less straightforward to distinguish between delayed clearance and premature reactivation. Nevertheless, the high frequency of Pol I transcripts observed in anaphase, also observed in HeLa cells, strongly supports a role for TTF2 in regulating Pol I activation during mitotic exit in these cells. Interestingly, prolonged mitotic arrest in HeLa cells leads to the disappearance of diffuse signals, as expected for failures in the clearance of Pol II transcripts. In sharp contrast, clustered signals remain even after long-term mitotic arrest (Fig S6). These findings suggest that in this cell type, TTF2-dependent control of Pol I activity may occur even at earlier mitotic stages, highlighting potential differences among cell types.

How TTF2 controls the timing of Pol I activation remains unknown. Clearance of engaged RNA polymerases is consistent with the established in vitro activity of TTF2, where it promotes displacement of both Pol I and II from DNA templates (Hara et al, 1999). However, regulation of Pol I may involve additional mechanisms, as Pol I, unlike Pol II, is not fully evicted from chromatin during mitosis (Scheer & Rose, 1984). It is therefore conceivable that TTF2 induces local changes in DNA organisation at rRNA loci, such as altered helical torsion characteristic of SNF2-like helicases (Dürr et al, 2006), thereby impairing efficient transcription initiation or elongation without complete displacement of Pol I. Still, we cannot exclude the possibility that the effects on Pol I result from an indirect effect on other Pol I regulators.

**Figure S7.**
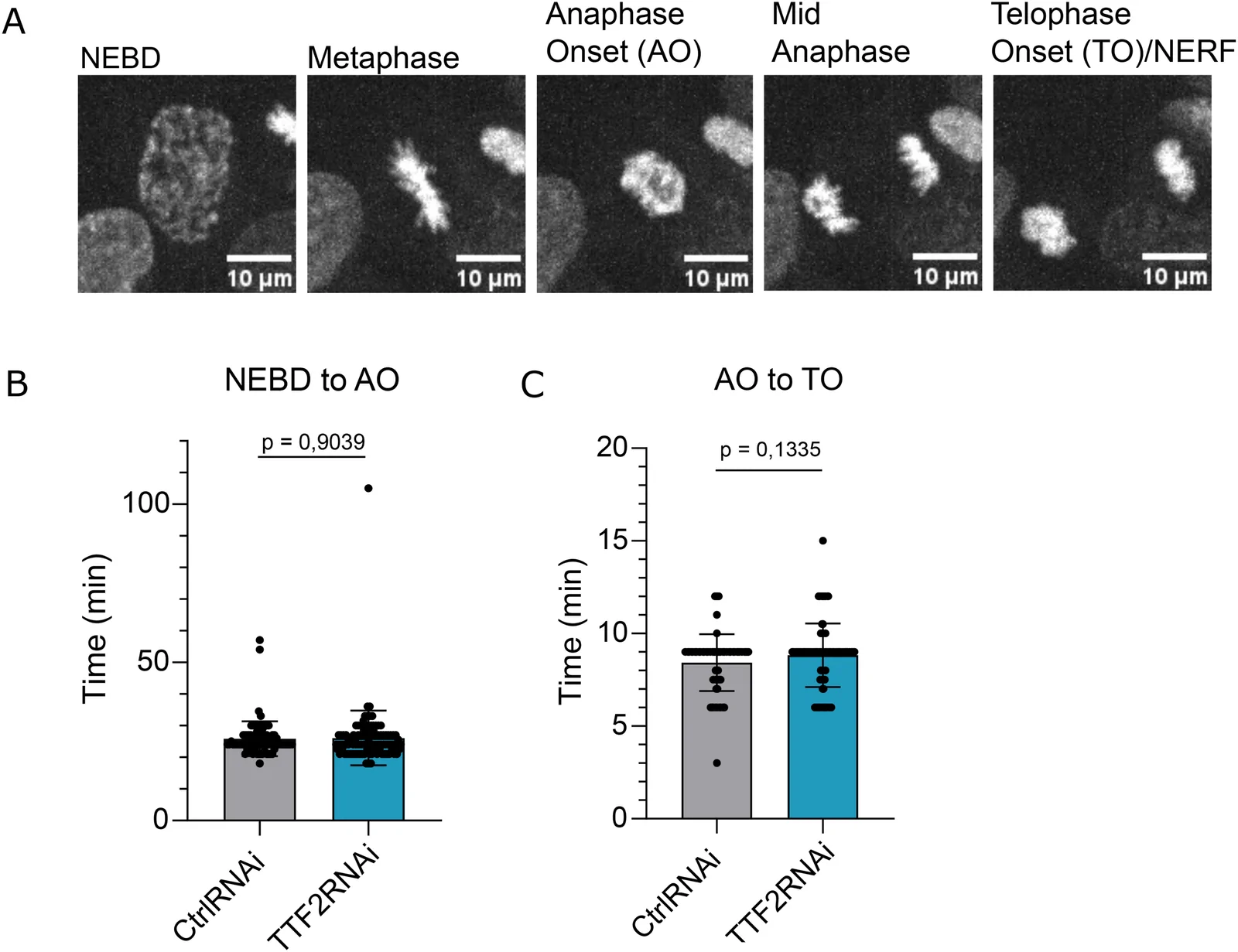
Mitotic timings are not altered upon TTF2 depletion in H9 human embryonic stem cells. **(A)** Representative mitotic stages of Control cells from live-cell imaging. **(B, C)** Quantification of the time from nuclear envelope breakdown (NEBD) to anaphase onset (AO) (B) and from AO to telophase onset (TO)/nuclear envelope reformation (NERF) (C) in Control and TTF2-depleted cells. Three independent experiments analysed. Each dot represents one cell; error bars indicate the mean ± SD. Statistical analysis was performed using the nonparametric Mann–Whitney test.

Our findings of premature Pol I–dependent nascent RNA synthesis at anaphase/telophase upon TTF2 depletion indicate that rRNA transcription can be uncoupled from cell cycle progression. This finding challenges prevailing models of Pol I regulation during mitotic exit. Canonical models propose that Pol I activation depends on reversal of Cdk1-mediated inhibitory phosphorylation events; consistent with this, inhibition of Cdk activity by roscovitine in mitotically arrested cells is sufficient to induce rRNA transcription (Sirri et al, 2000). More detailed in vitro studies have suggested that full Pol I activation requires phosphorylation of UBF by G1 cyclin–dependent mechanisms, a process thought to occur only in G1 (Heix, 1998; Klein & Grummt, 1999). Similarly other Pol I regulators, such as TBP, bind more strongly to rDNA loci during mitosis, and loss of this mitotic binding delays reactivation of Pol I transcription after cell division (Kwan et al, 2024). In contrast, our results support a model in which TTF2 provides a critical layer of repression restraining Pol I activity during mitosis. Notably, depletion of TTF2 in H9 cells induces premature Pol I–dependent transcription without obvious changes in the timing of mitotic progression or Cdk inactivation (e.g., no changes in mitotic progression or nuclear envelope reformation (NERF) (Fig S7A–C). This indicates that Pol I reactivation can occur in a mitotic context and positions TTF2 as a safeguard preventing untimely rRNA synthesis during mitotic exit.

Our findings also inform the temporal relationship between Pol I reactivation and early nucleolar reassembly. Notably, this process is factor specific. For nucleolin, we show that its recruitment can be anticipated by premature rRNA transcription, suggesting that nucleolin recruitment is also uncoupled from cell cycle control and is instead regulated by rRNA transcription onset and/or TTF2. In contrast to nucleolin, nucleophosmin is not detected at transcriptional sites during mitotic exit. These observations are in agreement with its late assembly upon mitotic exit (Leung et al, 2004; Fu et al, 2023).

We further demonstrate that TTF2 depletion is associated with defects in nucleolar integrity. It remains unclear whether the observed nucleolar defects are a direct cause of mistimed rRNA transcriptional reactivation or reflect additional roles of TTF2 in regulating Pol I. However, the fact that TTF2 binds to chromatin exclusively in mitosis (Jiang et al, 2004) strongly supports that nucleolar defects are a consequence of mitotic unscheduled rRNA transcription regulation. In either case, the aberrant nucleolar organisation upon loss of TTF2 likely reflects impaired nucleolar function, a phenotype commonly associated with cellular stress and disease states (Yang et al, 2018). Given the central role of Pol I transcription in ribosome biogenesis and cell growth, perturbation of this tightly regulated mitotic program may have broader consequences for cells’ proliferative capacity.

An important remaining question is whether similar mechanisms restrain Pol II reactivation during mitotic exit. Although our data robustly capture premature Pol I reactivation, the high dynamic range of rRNA synthesis provides a particularly sensitive readout of early transcriptional restart. In contrast, Pol II reactivation involves lower amplitude, gene-specific transcriptional events that may be more difficult to resolve during the rapid transitions of mitotic exit. It is therefore conceivable that transcriptional clearance mechanisms may also impact Pol II–mediated transcriptional restart in the subsequent cell cycle. Accordingly, previous studies have shown that increasing cohesin stability results in retention of elongating Pol II on mitotic chromatin and in gene expression defects after mitotic exit (Perea-Resa et al, 2020). However, because cohesin and its regulators play central roles in 3D genome organisation and transcriptional control throughout interphase (Liu et al, 2021), it remains difficult to disentangle direct effects of mitotic transcriptional perturbation from indirect consequences of altered chromatin architecture.

In summary, our work identifies TTF2 as a central regulator of transcriptional dynamics across mitosis, coordinating transcriptional shutdown at mitotic entry and reactivation at mitotic exit. Although governed by a shared regulatory factor, these processes proceed with distinct kinetics and involve different RNA polymerases, underscoring that transcriptional reactivation is not a simple reversal of mitotic silencing. Together, these findings establish mitosis as an actively regulated transcriptional transition and position TTF2 as a key integrator of transcriptional control and nucleolar organisation across cell division. Failures in mitotic transcriptional control may thus have broader implications for how transcriptional programs are improperly re-established during development or underlie nucleolar dysfunction.

## Materials and Methods

### Cell culture and maintenance

H9 and HeLa cell lines were grown at 37°C in 5% CO_2_ incubator. HeLa cells were grown in DMEM high glucose (L0103; Biowest) supplemented with 10% (vol/vol) foetal bovine serum (FBS) (Biowest) and 1% (vol/vol) penicillin–streptomycin (Gibco).

H9 cells were cultured in TeSR-E8 Basal Medium supplemented with TeSR-E8 25X supplement (Stem Cell Technologies) on vitronectin (Gibco)-coated 6-well plates. For cell maintenance, EDTA-based Gentle Cell Dissociation Agent TrypLE Express Enzyme (Gibco) was used and mechanical dissociation applied. For cell seeding and downstream experiment, cells were detached using TrypLE Express and plated in E8 Basal Medium containing Y-27632 (Rock) inhibitor.

### siRNA transfection for knockdown induction

RNAi was achieved by transfection with 200 ng/μl siRNA (EHU049891 for TTF2 and EHUFLUC as negative control), MISSION siRNA (Sigma-Aldrich) using Lipofectamine RNAi MAX (Invitrogen) and Opti-MEM (Gibco), according to the manufacturers’ protocol. Specificity of the TTF2 siRNA towards mitotic transcription retention was previously validated (Tovini et al, 2026 *Preprint*).

For HeLa cells, 5 × 10^4^ cells were seeded in each of 12-well plates on glass coverslips and knockdown performed for 48 h. For H9 cells, 1.2 × 10^5^ cells were seeded in each of 12-well plates and knockdown was performed in two consecutive rounds: the first one at the same time as cell seeding, followed by media replacement after 16 h (without Y-27632) and a second round of transfection after an additional 8 h, for a total of 48 h.

### Drug treatments

To arrest cells in metaphase, HeLa cells were treated with nocodazole (0.1 μg/ml) for the last 16 h, or with Apcin (12 μM) and proTAME (12 μM) for the last 4 h, of the 48-h knockdown period. Cells were then processed for EU incorporation.

### Nascent transcription detection and immunofluorescence

Labelling of nascent transcripts was performed by EU incorporation (1 mM, 30 min in media) followed by detection via ClickiT chemistry (ClickiT RNA Imaging Kits, C10329; Invitrogen) according to the manufacturer’s instructions. When combined with immunofluorescence, after Click-iT RNA reaction cells were washed with PBS and incubated with 3% BSA in PBS for 30 min for blocking. Cells were then incubated with primary antibodies for 1 h at room temperature (anti-nucleolin, ab136649; Abcam, 1:400; anti-nucleophosmin ab10530; Abcam, 1:1,000), followed by PBS washes and incubation with secondary antibodies (Goat anti-mouse Alexa Fluor 568 Invitrogen, 1:1,000). After wash, DNA was stained with Hoechst (1:1,000), and coverslips were mounted with Vectashield (Vector H-1000; Vector Laboratories).

### rRNA FISH

To detect rRNA, FISH probes were designed and produced by Molecular Instruments HCR (Table S1), and hybridization was performed according to the manufacturer’s directions. When used in combination with the EU Click-iT protocol, cells were fixed after EU incorporation and permeabilized overnight with 70% EtOH at −20°C. The following day, the Click-iT protocol was performed as above, followed by the RNA FISH staining.

Table S1. List of the FISH RNA target sites, number, and sequences of the RNA FISH probes (HCR Molecular Instruments) used.

### Western blotting

To confirm the efficient protein knockdown, cells were collected and stored at −80°C until usage or immediately lysed with RIPA buffer (25 mM Tris–HCl, pH 7.6, 150 mM NaCl, 1% NP-40, 1% sodium deoxycholate, 0.1% SDS) supplemented with protease inhibitor (PIER78429; Thermo Fisher Scientific). Samples were quantified with Bradford assay (Bio-Rad), loaded on a 4–12% Mini-PROTEAN TGX Precast Gels (Bio-Rad) and transferred to a nitrocellulose blot membrane. Western blot analysis was performed according to standard protocols using the following antibodies: anti-TTF2 (1:1,000, ab28030; Abcam); anti-GAPDH (1:2,000, 14C10; Cell Signalling Technology); anti-nucleolin (1:1,000, ab136649; Abcam); rabbit anti-sheep IgG (H&L) (DyLight 800 Conjugated, 1:10,000, 613-445-002; Rockland); goat anti-rabbit (IRDye 800CW, 1:10,000, 926-32211); donkey anti-mouse (IRDye 680LT, 1:10,000, 926-68022).

### Microscopy

Fixed samples were imaged on an Andor Dragonfly Spinning Disk confocal microscope equipped with Andor Sona sCMOS 4 MPix camera using a 1,600 × 1,350 FOV. 60× 1.2 NA water immersion objective was used.

For live-cell imaging, H9 and HeLa-FBL-mGFP cells were seeded in an 8-well Ibidi chamber and transfected as described above, maintaining the ratio of cells, media, and transfectant reagents. To determine mitotic timings, 48 h after transfection with Control and TTF2-specific siRNAs, H9 cells were incubated with SiR-DNA (Spyrochrome) in media for 30 min, washed and replaced with normal media, and imaged in a CO_2_ (5%) and T (37°C) controlled chamber. Cells were imaged with a 40× dry objective every 3 min for 8 h. For analysis of nucleolar dynamics, at 24 h after RNAi treatment, HeLa-FBL-mGFP were also incubated with SiR-DNA (Spyrochrome) in media for 30 min, washed and replaced with normal media, and imaged in a CO_2_ (5%) and T (37°C) controlled chamber. Cells were imaged with a 40× dry objective every 5 min for 8 h.

### Image analysis

Images were analysed using FIJI software, in z-stacks or max-intensity projections.

For the qualitative assessment of presence or absence of EU, a blind analysis was performed by attributing random file name to the acquired images. To discriminate chromatin-bound signals from the chromosome-periphery coating signal, images were analysed by examining each optical section throughout the z-stack.

For the quantitative imaging analysis of the nascent RNA on chromatin (EU) or rRNA (FISH probes), images were analysed after max-intensity z-projection. The signal from the channel of interest was measured using a mask based on DAPI threshold to delineate the chromatin area. Measurements were subtracted by background intensity (measured on an area outside of the cell).

For the qualitative assessment of EU co-localization with rRNA FISH probes (47S, 5′ETS, and 3′ETS), as well as with nucleolin and nucleophosmin, images were examined across z-stacks to distinguish chromatin-associated signals from signals coating the chromosome periphery. EU overlap with nucleolin and nucleophosmin was classified according to their chromatin distribution and spatial coincidence across the chromatin mass.

To analyse nucleoli distribution in fixed H9 and HeLa cell samples, the nucleolin marker signal was used as threshold to identify individual nucleoli, which were then selected manually and counted using a custom macro. In live-cell imaging, nucleoli number was determined using the fibrillarin marker and counted manually.

### Statistical analysis

Statistical analysis was performed using GraphPad Prism 9.0. or Jupyter Notebook specific statistics Python package. Detailed information on the statistical tests used is described in the figure legends.

For statistical analysis of the percentage of H9 and HeLa cells displaying different types of EU signal, cells at each mitotic stage were categorized into one of the three EU signal states (“none,” “diffuse,” or “clustered”) for Control and TTF2RNAi conditions. Firth’s penalized logistic regression (Benjamini–Hochberg corrected) was performed in Python (NumPy, SciPy) inside Jupyter Notebook.

For statistical analysis of the percentage of cells with EU signal in DMSO, TRP, and BMH treatments, cells were scored as signal-positive or signal-negative across four conditions (Control/TTF2 siRNA × DMSO/drug). Pairwise comparisons were tested using Firth’s penalized logistic regression (Benjamini–Hochberg corrected). Analysis was performed in Python (NumPy, SciPy) inside Jupyter Notebook.

To analyse the 5′ETS ad 3′ETS rRNA FISH probes results, cells were scored as signal-positive or signal-negative for 5′ETS and 3′ETS at each mitotic stage (early/mid/late anaphase, telophase), comparing Control and TTF2RNAi. Each comparison was tested using Firth’s penalized logistic regression. Analysis was performed in Python (NumPy, SciPy) using Jupyter Notebook.

For the nucleolar distribution analysis, nucleoli counts per cell were compared between Control and TTF2RNAi using a Poisson generalized linear model (GLM). To identify which specific nucleoli count categories drove this difference, follow-up Firth’s penalized logistic regressions were performed for each category (category versus all others), with *P*-values corrected for multiple testing using the Benjamini–Hochberg method. Analysis was performed in Python (NumPy, pandas, SciPy, and statsmodels), inside Jupyter Notebook.

## Supplementary Material

Reviewer comments

## Data Availability

All tools used in this project are either commercially available or will be made available upon request to RA Oliveira.
