# Supplementary material for: Transcription termination factor 2 (TTF2) prevents premature rRNA synthesis during mitotic exit
Source: Life Sci Alliance. 2026 Oct 1;9(12):e202603841. doi: 10.26508/lsa.202603841 (PMC13631568; doi:10.26508/lsa.202603841)
Supplement: Supplementary file 1 [file LSA-2026-03841_review_history.pdf]

Transcription Termination Factor 2 (TTF2) prevents premature rRNA synthesis during mitotic exit

Catarina Pedro, Laura Tovini, Catarina Peneda, Mia Krapinec, and Raquel Oliveira
DOI: https://doi.org/10.26508/lsa.202603841

Corresponding author(s): Raquel Oliveira, Católica Biomedical Research Centre

Review Timeline:

Submission Date:	2026-07-08
Editorial Decision:	2026-07-10
Revision Received:	2026-08-19
Editorial Decision:	2026-09-11
Revision Received:	2026-09-17
Accepted:	2026-09-21

Scientific Editor: Sarita Hebbar

Transaction Report:

(Note: With the exception of the correction of typographical or spelling errors that could be a source of ambiguity, letters and reports are not edited. The original formatting of letters and referee reports may not be reflected in this compilation.)

Please note that the manuscript was reviewed at *Review Commons* and these reports were taken into account in the decision-making process at *Life Science Alliance.*

Reviews

Review #1

1. Evidence, reproducibility and clarity:

In this paper by the Oliveira lab a new perspective on TTF2 function during mitosis is proposed. Known for its role to terminate transcription at mitotic onset, this paper further shows an exciting involvement of TTF2 to schedule timely rDNA transcription at mitotic exit. Moreover, this role is shown to have a clear importance in the structuration of nucleoli, since TTF2 depletion is associated with premature partial assembly of nucleoli on the mitotic chromosomes and, subsequently, to fragmented nucleoli in interphase. These conclusions, which are well supported by imaging data, are original, interesting and, in fact, largely unexpected.

The paper is very simple in its execution, based on siRNA depletion of TTF2 and monitoring of transcription by imaging using EU incorporation and rRNA-FISH, as well as nucleoli morphology and dynamics using immunostainings. Yet, it is well executed and has no major caveats. However, the authors should consider the following:

1. The Teves lab has shown that TBP is a key factor maintaining its binding at rDNA loci during mitosis, enabling a prompt reactivation of rRNA production in interphase (Kwan et al. RNA 2024). This paper should be discussed on the light of current findings.
2. In relation to the previous comment, I would strongly recommend the authors to analyse TBP-depleted cells, ideally using the line generated in the Teves lab, to address whether delayed rDNA transcription after mitosis leads to delayed nucleoli structuration. This assay would allow them to further confirm their model.
3. In addition, it would be important to test if in the absence of mitotic TBP, the depletion of TTF2 does also lead to mitotic transcription.

2. Significance:

Significance (Required)

Strengths: originality of the observation and simplicity of the experimental setup

Limitations: exclusively based on imaging data

Advance: completely unanticipated observation

Audience: general readers interested in gene regulation

My expertise: gene regulation through mitosis

3. How much time do you estimate the authors will need to complete the suggested revisions:

Estimated time to Complete Revisions (Required)

(Decision Recommendation)

Between 1 and 3 months

4. *Review Commons* values the work of reviewers and encourages them to get credit for their work. Select 'Yes' below to register your reviewing activity at Web of Science Reviewer Recognition Service (formerly Publons); note that the content of your review will not be visible on Web of Science.

Web of Science Reviewer Recognition

No

Review #2

1. Evidence, reproducibility and clarity:

Summary:

In this manuscript, the authors investigate the role of Transcription Termination Factor 2 (TTF2) in the regulation of

mitotic transcription. Using siRNA-mediated knockdown in two distinct human cell types combined with nascent RNA labeling (EU pulse), the authors identify an unexpected role for TTF2 in the timing of RNA Polymerase I (Pol I) reactivation following mitosis. The study suggests that this temporal misregulation may have downstream consequences for nucleolar morphology and function in interphase. The manuscript is well-written, and the figures are of high quality and clearly presented.

Major comments:

1. A primary limitation of the current study is that it does not deeply explore the underlying mechanism of the observed phenomenon. To strengthen the claims, the following points should be addressed:

1a. Directionality of Phenotype: In Page 9, the authors conclude that TTF2 depletion is linked to abnormal nucleolar organization during interphase. It remains unclear if this is a direct result of mitotic misregulation or an independent interphase effect. To distinguish between these possibilities, I suggest the following experiment: perform a mitotic shake-off early in the siRNA treatment (~24h), collect mitotic cells, and allow them to re-enter G1 to image for nascent RNAs and nucleolin. This would clarify if the mitotic defect precedes and causes the interphase morphology changes. Alternatively, the authors should state that their current study cannot distinguish between these two possibilities.

1b. Secondary Effects: The long duration of siRNA treatment (48h) raises the possibility that TTF2 knockdown misregulates the expression of other Pol I regulatory factors, leading to secondary effects. This limitation should be explicitly acknowledged in the Discussion.
2. The term "significant" is used throughout the manuscript without accompanying statistical testing.

2a. Please provide statistical analyses (e.g., p-values) for the average plots in Figures 1-3 to substantiate the findings.

2b. Where statistics are not performed, the language should be softened to "notable" or "observed increase" rather than "significant."
3. siRNA knockdowns are generally supported by quantification. Please provide the percentage reduction of the target protein by quantifying the blots provided in the supplemental figures.
4. To rule out the possibility that the increased nucleolin signal observed after TTF2 KD is simply due to higher protein abundance, the authors should perform a western blot to confirm that total nucleolin protein levels remain unchanged upon TTF2 depletion.

Minor comments:

1. The abstract and discussion refer to the role of TTF2 as a "conserved" process. As the study only tested human cell lines, "conserved" is technically inaccurate (as it implies evolutionary comparison). I recommend using "general" or "cell-type independent."
2. While the Methods section is detailed, the Results section would benefit from brief descriptions of the treatments to improve flow.

Example revision (Page 4): "...we treated two distinct cell lines with control and TTF2-specific siRNAs for 48 hours, followed by a 30-minute EU pulse to label nascent RNAs. Click chemistry and Hoechst labeling enabled 2-color imaging of mitotic chromosomes and nascent RNA..."
3. The data generally agree across both cell types; however, the presence of clustered signals in HeLa metaphase chromosomes is a notable divergence. It would be beneficial to include speculation in the Discussion on whether this represents a failure to silence Pol I transcription or an even earlier reactivation, and what this implies about a cancer cell line.

2. Significance:

Significance (Required)

General assessment:

The study is strong in its use of two different cell systems, providing confidence that the observed effects are not cell-line-specific. The figures are beautifully presented and the writing is clear. The primary limitation is the lack of mechanistic depth regarding how TTF2 specifically interfaces with the Pol I machinery compared to its known roles with Pol II.

Advance:

This work reports a previously unrecognized role for TTF2 in the temporal control of Pol I reactivation. While TTF2 is well-known for its role in terminating transcription and facilitating Pol II release during mitosis, its specific influence on the nucleolar transcription cycle provides a new perspective on how cells transition out of the mitotic state.

Audience:

This research will be of interest to researchers in the fields of gene regulation, the cell cycle, and nucleolar biology. Because it touches on the fundamental process of how transcriptional machinery is reset after cell division, it has implications for the broader basic research community interested in epigenetic memory and cellular identity.

3. How much time do you estimate the authors will need to complete the suggested revisions:

Estimated time to Complete Revisions (Required)

(Decision Recommendation)

Between 1 and 3 months

4. *Review Commons* values the work of reviewers and encourages them to get credit for their work. Select 'Yes' below to register your reviewing activity at [Web of Science Reviewer Recognition Service]() (formerly Publons); note that the content of your review will not be visible on Web of Science.

Web of Science Reviewer Recognition

Yes

1. General Statements

We were very pleased with the positive comments about our manuscript, acknowledging the importance and novelty of our results, uncovering a novel role for TTF2 in the control of rRNA transcriptional onset at mitotic exit.

We are also thankful for the constructive criticisms, which we plan to address in the full version, as detailed in section 2.
A few of the suggested experiments, particularly regarding the role of other players and deeper mechanistic insights, are beyond the scope of the current paper (see details in section 4). We, however, agree that dissecting further whether the nucleolar defects are a direct consequence of mitotic mis-timed activation or, alternatively, reflect an additional role of TTF2 in nucleolar structure during interphase would significantly strengthen our manuscript. Details on our plans to dissect this issue and other minor points raised are outlined in section 2.

2. Description of the planned revisions

Reviewer #1 (Evidence, reproducibility and clarity (Required)):

Comment: In this paper by the Oliveira lab a new perspective on TTF2 function during mitosis is proposed. Known for its role to terminate transcription at mitotic onset, this paper further shows an exciting involvement of TTF2 to schedule timely rDNA transcription at mitotic exit. Moreover, this role is shown to have a clear importance in the structuration of nucleoli, since TTF2 depletion is associated with premature partial assembly of nucleoli on the mitotic chromosomes and, subsequently, to fragmented nucleoli in interphase. These conclusions, which are well supported by imaging data, are original, interesting and, in fact, largely unexpected.

The paper is very simple in its execution, based on siRNA depletion of TTF2 and monitoring of transcription by imaging using EU incorporation and rRNA-FISH, as well as nucleoli morphology and dynamics using immunostainings. Yet, it is well executed and has no major caveats.

We thank the reviewer for the positive statements about our work.

Comment:

However, the authors should consider the following:

Revision Plan

1/ The Teves lab has shown that TBP is a key factor maintaining its binding at rDNA loci during mitosis, enabling a prompt reactivation of rRNA production in interphase (Kwan et al. RNA 2024). This paper should be discussed on the light of current findings.

We will extend the discussion to include the role of TBP in rRNA reactivation at mitotic exit.

Reviewer #2 (Evidence, reproducibility and clarity (Required)):

1. EVIDENCE, REPRODUCIBILITY AND CLARITY

Summary:

In this manuscript, the authors investigate the role of Transcription Termination Factor 2 (TTF2) in the regulation of mitotic transcription. Using siRNA-mediated knockdown in two distinct human cell types combined with nascent RNA labeling (EU pulse), the authors identify an unexpected role for TTF2 in the timing of RNA Polymerase I (Pol I) reactivation following mitosis. The study suggests that this temporal misregulation may have downstream consequences for nucleolar morphology and function in interphase. The manuscript is well-written, and the figures are of high quality and clearly presented.

We thank the reviewer for the positive statements about our work.

Major comments:

1. A primary limitation of the current study is that it does not deeply explore the underlying mechanism of the observed phenomenon. To strengthen the claims, the following points should be addressed:

1a. Directionality of Phenotype: In Page 9, the authors conclude that TTF2 depletion is linked to abnormal nucleolar organization during interphase. It remains unclear if this is a direct result of mitotic misregulation or an independent interphase effect. To distinguish between these possibilities, I suggest the following experiment: perform a mitotic shake-off early in the siRNA treatment (~24h), collect mitotic cells, and allow them to re-enter G1 to image for nascent RNAs and nucleolin. This would clarify if the mitotic defect precedes and causes the interphase morphology changes. Alternatively, the authors should state that their current study cannot distinguish between these two possibilities.

We fully agree that providing additional evidence supporting the directionality between rRNA mis-timed reactivation and nucleolar defects is an important aspect that should be substantiated. For this, we plan to perform the experiments similar to the ones suggested by the referee:

1. *RNAi for a shorter time-period (24hr). – As the reviewer suggest, we anticipate that shortening the RNAi-depletion will provide a separation of visible phenotypes to cells that have only recently undergone mitosis (G1) versus cells that have undergone cell division before efficient TTF2 depletion. We think this analysis can be done without cell sorting as G1 vs G2 cells can be distinguished by nuclear size and/or cell cycle markers. Nucleoli will be evaluated as in our current Fig. 5.*
2. *We will additionally perform live-cell imaging using nucleolar markers (cell lines from S. Cuylen-Haering Lab have already been obtained for this analysis) to evaluate the kinetics of nucleolar defects in relation to mitotic exit.*

Revision Plan

If this approach fails to substantiate the order of events, or provides evidence for a direct role of TTF2 in interphase (not our prime hypothesis, but equally interesting), we will update the discussion accordingly, emphasising the noted caveat.

1b. Secondary Effects: The long duration of siRNA treatment (48h) raises the possibility that TTF2 knockdown misregulates the expression of other Pol I regulatory factors, leading to secondary effects. This limitation should be explicitly acknowledged in the Discussion.

We trust the experiments planned to address the point above will also minimise this valid caveat. We will nevertheless also address this caveat explicitly in the discussion.

2. The term "significant" is used throughout the manuscript without accompanying statistical testing.

2a. Please provide statistical analyses (e.g., p-values) for the average plots in Figures 1-3 to substantiate the findings.

We will include statistical analysis for all the experiments.

2b. Where statistics are not performed, the language should be softened to "notable" or "observed increase" rather than "significant."

We will soften the wording for any claim that is not substantiated by statistics (which will be limited to Fig. 2I, where no equivalent control can be used for statistical comparisons).

3. siRNA knockdowns are generally supported by quantification. Please provide the percentage reduction of the target protein by quantifying the blots provided in the supplemental figures.

Figure S1 will be updated to include quantifications of RNAi efficiency derived from 3 independent experiments, for both H9 and HeLa cells.

4. To rule out the possibility that the increased nucleolin signal observed after TTF2 KD is simply due to higher protein abundance, the authors should perform a western blot to confirm that total nucleolin protein levels remain unchanged upon TTF2 depletion.

A new supplementary figure will be added to include quantifications of total levels of nucleolin, as suggested.

Minor comments:

1. The abstract and discussion refer to the role of TTF2 as a "conserved" process. As the study only tested human cell lines, "conserved" is technically inaccurate (as it implies evolutionary comparison). I recommend using "general" or "cell-type independent."

We agree with the reviewer. We will rephrase the wording to avoid the use of "conserved" in this context.

2. While the Methods section is detailed, the Results section would benefit from brief descriptions of the treatments to improve flow.

Example revision (Page 4): "...we treated two distinct cell lines with control and TTF2-specific siRNAs for 48 hours, followed by a 30-minute EU pulse to label nascent RNAs. Click chemistry and Hoechst labeling enabled 2-color imaging of mitotic chromosomes and nascent RNA...".

We will improve the results description to better clarify the experimental layout.

3. The data generally agree across both cell types; however, the presence of clustered signals in HeLa metaphase chromosomes is a notable divergence. It would be beneficial to include speculation in the Discussion on whether this represents a failure to silence Pol I transcription or an even earlier reactivation, and what this implies about a cancer cell line.

We agree with the reviewer's point. In asynchronous cells, the known role for TTF2 in transcript clearance made it difficult to establish the nature of the observed signals in metaphase (perdurance vs reactivation). To attempt to disentangle this issue, we will address EU incorporation in response to prolonged metaphase arrest. We anticipate that if the metaphase clustered signals are a result of failure to clear previously active Pol I molecules, the signal should lower over time (similarly to mRNA signals). If, in contrast, the clustered signals persist, it suggests that it likely corresponds to premature reactivation, even in metaphase. If conclusive, these results will be included and possible reasons for the observed differences (e.g. cancer state) will be included in the discussion, accordingly.

3. Description of the revisions that have already been incorporated in the transferred manuscript

4. Description of analyses that authors prefer not to carry out

Reviewer 1 Comments:

2/ In relation to the previous comment, I would strongly recommend the authors to analyse TBP-depleted cells, ideally using the line generated in the Teves lab, to address whether delayed rDNA transcription after mitosis leads to delayed nucleoli structuration. This assay would allow them to further confirm their model.

3/ In addition, it would be important to test if in the absence of mitotic TBP, the depletion of TTF2 does also lead to mitotic transcription.

We agree that full dissection of the mechanism by which TTF2 depletion impacts Pol I reactivation would require analysis as suggested by the reviewers for previously known co-factors. This includes TBP, but also SL1, UBF, all of which with previously known roles in regulating Pol I reactivation at mitotic exit (and probably many others yet to be uncovered). This analysis, however, is beyond the scope of the current manuscript.

July 10, 2026

Re: Life Science Alliance manuscript #LSA-2026-03841-T

Dr. Raquel A Oliveira
Católica Biomedical Research Centre
Católica Biomedical Research Centre
GIMM campus
Rua da Quinta Grande, 6
Lisboa 2780-156
Portugal

Dear Dr. Oliveira,

Thank you for submitting your manuscript entitled "TTF2 prevents premature rRNA synthesis during mitotic exit" to Life Science Alliance.

We invite to submit a manuscript revised according to your submitted revision plan.

To upload the revised version of your manuscript, please log in to your account: https://lsa.msubmit.net/cgi-bin/main.plex

You will be guided to complete the submission of your revised manuscript and to fill in all necessary information. Please get in touch in case you do not know or remember your login name.

The typical timeframe for revisions is three months. Please note that papers are generally considered through only one revision cycle, so strong support from the referees on the revised version is needed for acceptance.

While you are revising your manuscript, please also attend to the below editorial points to help expedite the publication of your manuscript. Please direct any editorial questions to the journal office. When submitting the revision, please include a letter addressing the reviewers' comments point by point.

Thank you for this interesting contribution to Life Science Alliance. We hope that the comments below will prove constructive as your work progresses, and we are looking forward to receiving your revised manuscript.

Sincerely,

Sarita Hebbar, PhD
Scientific Editor
Life Science Alliance
http://www.lsajournal.org

A. THESE ITEMS ARE REQUIRED FOR REVISIONS

-- A letter addressing the reviewers' comments point by point.

-- An editable version of the final text (.DOC or .DOCX) is needed for copyediting (no PDFs).

-- High-resolution figure, supplementary figure and video files uploaded as individual files: See our detailed guidelines for preparing your production-ready images, https://www.life-science-alliance.org/authors

-- Summary blurb (enter in submission system): A short text summarizing in a single sentence the study (max. 200 characters including spaces). This text is used in conjunction with the titles of papers, hence should be informative and complementary to the title and running title. It should describe the context and significance of the findings for a general readership; it should be written in the present tense and refer to the work in the third person. Author names should not be mentioned.

-- By submitting a revision, you attest that you are aware of our payment policies found here: https://www.life-science-alliance.org/copyright-license-fee

B. MANUSCRIPT ORGANIZATION AND FORMATTING:

Full guidelines are available on our Instructions for Authors page, https://www.life-science-alliance.org/authors

We encourage our authors to provide original source data, particularly uncropped/-processed electrophoretic blots and spreadsheets for the main figures of the manuscript. If you would like to add source data, we would welcome one PDF/Excel-file per figure for this information. These files will be linked as supplementary "Source Data" files.

IMPORTANT: It is Life Science Alliance policy that if requested, original data images must be made available. Failure to provide original images upon request will result in unavoidable delays in publication. Please ensure that you have access to all original microscopy and blot data images before submitting your revision.

Response to reviewers' comments - RC- 2026-03510

General comments:

We were very pleased with the positive comments about our manuscript, acknowledging the importance and novelty of our results, uncovering a novel role for TTF2 in the control of rRNA transcriptional onset at mitotic exit.

We are also thankful for the constructive criticisms, which we now have fully addressed, as detailed below. We trust these changes strengthen the conclusions of the manuscript.

Reviewer #1 (Evidence, reproducibility and clarity (Required)):

Comment: In this paper by the Oliveira lab a new perspective on TTF2 function during mitosis is proposed. Known for its role to terminate transcription at mitotic onset, this paper further shows an exciting involvement of TTF2 to schedule timely rDNA transcription at mitotic exit. Moreover, this role is shown to have a clear importance in the structuration of nucleoli, since TTF2 depletion is associated with premature partial assembly of nucleoli on the mitotic chromosomes and, subsequently, to fragmented nucleoli in interphase. These conclusions, which are well supported by imaging data, are original, interesting and, in fact, largely unexpected.

The paper is very simple in its execution, based on siRNA depletion of TTF2 and monitoring of transcription by imaging using EU incorporation and rRNA-FISH, as well as nucleoli morphology and dynamics using immunostainings. Yet, it is well executed and has no major caveats.

We thank the reviewer for the positive statements about our work.

Comment:

However, the authors should consider the following:

1/ The Teves lab has shown that TBP is a key factor maintaining its binding at rDNA loci during mitosis, enabling a prompt reactivation of rRNA production in interphase (Kwan et al. RNA 2024). This paper should be discussed on the light of current findings.

2/ In relation to the previous comment, I would strongly recommend the authors to analyse TBP-depleted cells, ideally using the line generated in the Teves lab, to address whether delayed rDNA transcription after mitosis leads to delayed nucleoli structuration. This assay would allow them to further confirm their model.

3/ In addition, it would be important to test if in the absence of mitotic TBP, the depletion of TTF2 does also lead to mitotic transcription.

We agree that full dissection of the mechanism by which TTF2 depletion impacts Pol I reactivation would require analysis as suggested by the reviewer, testing the contribution of previously known co-factors. This includes TBP, but also SL1, UBF, all of which with previously known roles in regulating Pol I reactivation at mitotic exit (and probably many others yet to be uncovered). This analysis, however, is beyond the scope of the current manuscript.

Nevertheless, we extended the introduction and discussion to include the role of TBP in rRNA reactivation at mitotic exit. You can now read:

(introduction - page 4)

Furthermore, the TATA-binding protein (TBP) has also been shown to bookmark rDNA loci during mitosis, a binding event which is necessary for proper and timely reactivation of Pol I transcription after mitosis (Kwan et al., 2024).

(discussion- page 12)

Similarly, other Pol I regulators, such as TBP, bind more strongly to rDNA loci during mitosis, and loss of this mitotic binding delays reactivation of Pol I transcription after cell division (Kwan et al., 2024).

Reviewer #2 (Evidence, reproducibility and clarity (Required)):

1. EVIDENCE, REPRODUCIBILITY AND CLARITY

Summary:

In this manuscript, the authors investigate the role of Transcription Termination Factor 2 (TTF2) in the regulation of mitotic transcription. Using siRNA-mediated knockdown in two distinct human cell types combined with nascent RNA labeling (EU pulse), the authors identify an unexpected role for TTF2 in the timing of RNA Polymerase I (Pol I) reactivation following mitosis. The study suggests that this temporal misregulation may have downstream consequences for nucleolar morphology and function in interphase. The manuscript is well-written, and the figures are of high quality and clearly presented.

We thank the reviewer for the positive statements about our work.

Major comments:

1. A primary limitation of the current study is that it does not deeply explore the underlying mechanism of the observed phenomenon. To strengthen the claims, the following points should be addressed:

1a. Directionality of Phenotype: In Page 9, the authors conclude that TTF2 depletion is linked to abnormal nucleolar organization during interphase. It remains unclear if this is a direct result of mitotic misregulation or an independent interphase effect. To distinguish between these possibilities, I suggest the following experiment: perform a mitotic shake-off early in the siRNA treatment (~24h), collect mitotic cells, and allow them to re-enter G1 to image for nascent RNAs and nucleolin. This would clarify if the mitotic defect precedes and causes the interphase morphology changes. Alternatively, the authors should state that their current study cannot distinguish between these two possibilities.

We fully agree that providing additional evidence supporting the directionality between rRNA mis-timed reactivation and nucleolar defects is an important aspect that needed to be substantiated. For this, we have now included the following experiments:

1. RNAi for a shorter time-period (24hr). – As the reviewer suggests, shortening the RNAi-depletion provides a separation of visible phenotypes to cells that have only recently undergone mitosis (G1) versus cells that have undergone cell division before efficient TTF2 depletion. To avoid the experimental difficulties associated with cell sorting, we have instead used nuclear size as a proxy for cell cycle stage. In accordance with a causal link, we observed that 24 hrs after depletion, nucleolar numbers are increased in smaller cells (G1/early S) but not in G2 (larger cells). In contrast, 48 hrs after TTF2 depletion nucleolar differences can be found across all nuclear sizes. These new results are now presented in **Supplementary figure 5** and the corresponding text reads (page 10):

   To confirm that increased nucleolar number is a consequence of mis-timed rRNA reactivation at mitotic exit, we repeated this analysis in TTF2 siRNA-treated H9 cells 24 hours after transfection (70% depletion of TTF2 protein levels, Supplementary Fig 5C,D). We reasoned that in this experimental condition, G1/early S-phase cells would represent cells that have undergone mitosis in the absence of TTF2. In contrast, late S/G2 cells are enriched in

cells which have undergone their last mitosis with sufficient TTF2 levels. For this distinction, we used nuclear size as a proxy for cell cycle stage. To better analyse these distributions, we divided 24-hour treated cells according to the nuclear size below or above the mean population size (214 ± SD , n= 510 cells). While the nucleolar area normalised to the respective nuclear size, remained unaltered (Supplementary Fig. 5G), we observed that smaller TTF2-depleted cells (likely representing G1/early S-phase) have a nucleolar distribution significantly different from their respective controls (Supplementary Fig. 5E). In contrast, larger cells do not show significant differences (Supplementary Fig. 5F). 48 hours after TTF2 depletion, both small and large cells show a significantly different distribution, as expected for a less temporally-resolved experimental condition (Supplementary Fig. 5 A,B).

2. In HeLa cells we could not perform similar analysis as the genetic instability/aneuploidy state in these cells precludes a clear correlation between nuclear size and cells cycle stage. To circumvent this limitation, we have performed live-cell imaging using nucleolar markers (HeLa cell lines from S. Cuylen-Haering Lab have already been obtained for this analysis, Schichler et al 2026) to evaluate the kinetics of nucleolar defects in relation to mitotic entry (G2) and mitotic exit (G1). This analysis revealed that 24-hour after TTF2 depletion, differences in nucleolar numbers could only be found in G1 but not G2 cells, as expected for a mitosis-related phenotype. These new results are now presented in **Supplementary figure 5** and the corresponding text reads (page 10):

To test if nucleolar number increase is a consequence of mitotic-related defects, we used live cell imaging in HeLa cells expressing GFP-tagged fibrillarin, an important nucleolar component (Schichler et al., 2026) to evaluate nucleolar number in G2 and G1 stages, using 24 hours TTF2 depletion to reduce cumulative effects (Supplementary Fig 5H, I). This analysis reveals that TTF2-depleted G2 cells do not show a statistically different nucleolar number, consistent with the notion that in their previous mitosis, TTF2 levels were likely unperturbed. In sharp contrast, early G1 cells (1hr, 2hr and 3 hr post anaphase onset), show significantly higher nucleolar numbers after TTF2 depletion (Fig. 5 H, I).

1b. Secondary Effects: The long duration of siRNA treatment (48h) raises the possibility that TTF2 knockdown misregulates the expression of other Pol I regulatory factors, leading to secondary effects. This limitation should be explicitly acknowledged in the Discussion.

We trust the experiments addressing the point above, showing a clear effect by 24 hours after siRNA treatment, also minimise this valid caveat. We have nevertheless also addressed this caveat explicitly in the discussion (page 12):

We cannot exclude the possibility that the effects on Pol I result from an indirect effect on other Pol I regulators.

2. The term "significant" is used throughout the manuscript without accompanying statistical testing.

2a. Please provide statistical analyses (e.g., p-values) for the average plots in Figures 1-3 to substantiate the findings.

2b. Where statistics are not performed, the language should be softened to "notable" or "observed increase" rather than "significant."

We have substantiated the claims by including statistical analysis for all experiments. The only exception is Fig. 2L, where no equivalent control can be used for statistical comparisons. The description of these findings, however, does not use the word “significant”.

3. siRNA knockdowns are generally supported by quantification. Please provide the percentage reduction of the target protein by quantifying the blots provided in the supplemental figures.

Supplementary Figure 1 was updated to include quantifications of RNAi efficiency derived from 3 independent experiments, for both H9 and HeLa cells, at 48 hrour after depletion (the main time-point used in this study). We additionally included WBs and quantifications for 24- hr depletion in Supplementary Figure 5.

4. To rule out the possibility that the increased nucleolin signal observed after TTF2 KD is simply due to higher protein abundance, the authors should perform a western blot to confirm that total nucleolin protein levels remain unchanged upon TTF2 depletion.

We have included a new figure (Supplementary Figure 4) with quantifications of total levels of nucleolin, as suggested, which revealed no significant changes. These findings are described in the text as follows (page 9):

Notably, these findings are not attributable to increased protein levels, as the most commonly described forms of nucleolin protein (76, 100, and 110 kDa) remain unchanged following TTF2 depletion (Supplementary Fig. 4A, B).

Minor comments:

1. The abstract and discussion refer to the role of TTF2 as a "conserved" process. As the study only tested human cell lines, "conserved" is technically inaccurate (as it implies evolutionary comparison). I recommend using "general" or "cell-type independent."

We agree with the reviewer. We have rephrased the wording to avoid the use of "conserved" in this context.

2. While the Methods section is detailed, the Results section would benefit from brief descriptions of the treatments to improve flow.

Example revision (Page 4): "...we treated two distinct cell lines with control and TTF2-specific siRNAs for 48 hours, followed by a 30-minute EU pulse to label nascent RNAs. Click chemistry and Hoechst labeling enabled 2-color imaging of mitotic chromosomes and nascent RNA...".

We have expanded the results description to better clarify the experimental layout.

3. The data generally agree across both cell types; however, the presence of clustered signals in HeLa metaphase chromosomes is a notable divergence. It would be beneficial to include speculation in the Discussion on whether this represents a failure to silence Pol I transcription or an even earlier reactivation, and what this implies about a cancer cell line.

We agree with the reviewer's point. In asynchronous cells, the known role for TTF2 in transcript clearance made it difficult to establish the nature of the observed signals in metaphase (perdurance vs reactivation). To attempt to disentangle this issue, we have experimentally addressed EU incorporation in response to prolonged metaphase arrest. We reasoned that if the metaphase clustered signals are a result of failure to clear previously active Pol I molecules, the signal should lower over time (similarly to mRNA signals). If, in contrast, the clustered signals persist, it suggests that it likely corresponds to premature reactivation, even in metaphase. Our results indicate that in HeLa cells, prolonged metaphase arrest leads to the disappearance of most diffused signals, but clustered EU-labelled foci remain even after overnight arrest. These findings are more consistent with continued activation (even in metaphase) rather than defective clearance. We have included these findings in Supplementary figure 6 and described in the discussion, as follows (page 11):

Interestingly, prolonged mitotic arrest in HeLa cells leads to the disappearance of diffuse signals, as expected for failures in the clearance of Pol II transcripts. In sharp contrast, clustered signals remain even after long-term mitotic arrest (Supplementary Fig. 6A). These findings suggest that in this cell type, TTF2-dependent control of Pol I activity may occur even at earlier mitotic stages, highlighting potential differences among cell types.

We have refrained from further discussion on whether this relates to the cancer state of these cells, as the number of cancer vs non-cancer cell types does not allow any solid speculation.

September 11, 2026

RE: Life Science Alliance Manuscript #LSA-2026-03841-TR

Dr. Raquel A Oliveira
Católica Biomedical Research Centre
Católica Biomedical Research Centre
GIMM campus
Rua da Quinta Grande, 6
Lisboa 2780-156
Portugal

Dear Dr. Oliveira,

Thank you for submitting your revised manuscript entitled "TTF2 prevents premature rRNA synthesis during mitotic exit".

In accordance with our offer, your revised manuscript was sent back to Reviewer 2 for evaluation and their comments are appended below. As you will note, Reviewer 2 has acknowledged the improvement in your manuscript.

In line with the reviewer's support, we would be happy to publish your paper in Life Science Alliance pending final revisions necessary to meet our formatting guidelines.

MANUSCRIPT ORGANIZATION AND FORMATTING:

To avoid unnecessary delays in the acceptance and publication of your paper, please read the following information carefully. Full guidelines are available on our Instructions for Authors page, https://www.life-science-alliance.org/authors

Submission of a paper that does not conform to Life Science Alliance guidelines will delay the acceptance of your manuscript.

-We encourage you to include the full name for TTF2 in the manuscript title.
-Please include Supplemental Table 1 (mentioned on Page 16)
-Please upload your main manuscript text as an editable doc file.
-Please be sure that all authors are mentioned in the Authors' contribution section...Mia Maesano Krapine is missing.
-Please use the [10 author names et al.] format in your references (i.e., limit the author names to the first 10).
-Please remove figure legends from the figures... they should appear only in the manuscript file.
-Since Figures S3 and S6 have only one panel, it is unnecessary to label it as A. Please remove it from the figures, their legends, and call-outs in the manuscript text.
-Please add callouts for Figures S1A-C; S2A-B and S7A-C to your main manuscript text.
-Please be sure that the authorship listing and order is correct

We encourage our authors to provide original source data, particularly uncropped/-processed electrophoretic blots and spreadsheets for the main figures of the manuscript. If you would like to add source data, we would welcome one PDF/Excel-file per figure for this information. These files will be linked as supplementary "Source Data" files.

It is Life Science Alliance policy that if requested, original data images must be made available to the editors. Failure to provide original images upon request will result in unavoidable delays in publication. Please ensure that you have access to all original data images prior to final submission.

If you are planning a press release on your work, please inform us immediately to allow informing our production team and scheduling a release date.

We welcome submissions of potential cover images for the issue of LSA in which your work would appear. If you have high quality images associated with this work, please feel free to email these, with a caption, to the journal office.

LSA encourages authors to provide a 30-60 second video where the study is briefly explained. These videos will be appear embedded with the manuscript online at Life Science Alliance, and on social media to promote the published paper and authors (for examples, see https://docs.google.com/document/d/1-UWCfbE4pGcDdcgzcmiuJl2XMBJnxKYeqRvLLrLSo8s/edit?usp=sharing). Corresponding or first-authors are welcome to submit the video. Please submit only one video per manuscript. The video can be emailed to contact@life-science-alliance.org

FINAL FILES:

To upload the final version of your manuscript, please log in to your account: https://lsa.msubmit.net/cgi-bin/main.plex
You will be guided to complete the submission of your revised manuscript and to fill in all necessary information. Please get in touch in case you do not know or remember your login name.

The following items are required for acceptance.

-- An editable version of the final text (.DOC or .DOCX) is needed for copyediting (no PDFs).

-- High-resolution figure, supplementary figure and video files uploaded as individual files: See our detailed guidelines for preparing your production-ready images, https://www.life-science-alliance.org/authors

-- Summary blurb (enter in submission system): A short text summarizing in a single sentence the study (max. 200 characters including spaces). This text is used in conjunction with the titles of papers, hence should be informative and complementary to the title. It should describe the context and significance of the findings for a general readership; it should be written in the present tense and refer to the work in the third person. Author names should not be mentioned.

The license to publish form must be signed before your manuscript can be sent to production. A link to the license to publish form will be available to the corresponding author only. Please take a moment to check your funder requirements.

Reviews, decision letters, and point-by-point responses associated with peer-review at Life Science Alliance will be published, alongside the manuscript. If you do want to opt out of having the reviewer reports and your point-by-point responses displayed, please let us know immediately.

Thank you for your attention to these final processing requirements. Please revise and format the manuscript and upload materials as soon as you are able.

Thank you for this interesting contribution to the literature. We look forward to publishing your paper in Life Science Alliance.

Sincerely,

Sarita Hebbar, PhD
Scientific Editor
Life Science Alliance
http://www.lsajournal.org

Reviewer #2 (Comments to the Authors (Required)):

The authors have made great effort in addressing all of my previous (reviewer 2) comments. I believe the manuscript is substantially improved and is ready for publication.

September 21, 2026

RE: Life Science Alliance Manuscript #LSA-2026-03841-TRR

Dr. Raquel A Oliveira
Católica Biomedical Research Centre
Católica Biomedical Research Centre GIMM campus
Rua da Quinta Grande, 6
Lisboa 2780-156
Portugal

Dear Dr. Oliveira,

Thank you for submitting your Research Article entitled "Transcription Termination Factor 2 (TTF2) prevents premature rRNA synthesis during mitotic exit". It is a pleasure to let you know that your manuscript is now accepted for publication in Life Science Alliance. Congratulations on this interesting work.

The final published version of your manuscript will be deposited by us to PubMed Central upon publication.

Your manuscript will now progress through copyediting and proofing. It is journal policy that authors provide original data upon request.

Your article will publish open access upon publication under a CC-BY license.

Reviews, decision letters, and point-by-point responses associated with peer-review at Life Science Alliance will be published, alongside the manuscript. If you do want to opt out of having the reviewer reports and your point-by-point responses displayed, please let us know immediately.

IMPORTANT: If you will be unreachable at any time, please provide us with the email address of an alternate author. Failure to respond to routine queries may lead to unavoidable delays in publication.

Scheduling details will be available from our production department. You will receive proofs shortly before the publication date. Only essential corrections can be made at the proof stage so if there are any minor final changes you wish to make to the manuscript, please let the journal office know now.

DISTRIBUTION OF MATERIALS:
Authors are required to distribute freely any materials used in experiments published in Life Science Alliance. Authors are encouraged to deposit materials used in their studies to the appropriate repositories for distribution to researchers.

You can contact the journal office with any questions, contact@life-science-alliance.org

Again, congratulations on a very nice paper. I hope you found the review process to be constructive and are pleased with how the manuscript was handled editorially. We look forward to future exciting submissions from your lab.

Sincerely,

Sarita Hebbar, PhD
Scientific Editor
Life Science Alliance
http://www.lsajournal.org
