# Supplementary material for: Transcription termination factor 2 (TTF2) prevents premature rRNA synthesis during mitotic exit
Source: Life Sci Alliance. 2026 Oct 1;9(12):e202603841. doi: 10.26508/lsa.202603841 (PMC13631568; doi:10.26508/lsa.202603841)
Supplement: Supplementary file 2 [file LSA-2026-03841_TableS1.docx]

**Table S1 – List of the FISH RNA target sites, number and sequences of the RNA FISH probes (HCR Molecular Instruments) used.**

| Target | Number of Probes | Probe-binding Sequences |
| --- | --- | --- |
| 5'ETS | 1 | gCTgACACgCTgTCCTCTggCgACCTgTCgCTggAgAggTTgggCCTCCggA |
|  | 2 | AgCCgCCTgCCggggCCCgCgggCCTgCTgTTCTCTCgCgCgTCCgAgCgTC |
|  | 3 | gTCTCCgCggggTTgTCCgCCgCCCCTTCCCCggAgTggggggTTggCCggA |
|  | 4 | CCCCTCgTCTCTCCTCTCCCCgCCCgCCggCggTgCgTgTgggAAggCgTgg |
| 3'ETS | 1 | TCCgggCCgggACggggTCCggggAgCgTggTTTgggAgCCgCggAggCggC |
|  | 2 | CCgCTTCTTCggTTCCCgCCTCCTCCCCgTTCACCgCCggggCggCTCgTCC |
| 47S pre-Rrna | 1 | CCTTgCggTgCTCCTggAgCgCTCCgggTTgTCCCTCAggTgCCCgAggCCg |
|  | 2 | CCgCTCCCgTgCCgAgTCgTgACCggTgCCgACgACCgCgTTTgCgTggCAC |
|  | 3 | TCCgggggTCggCCTgCggCgCgTgCgggggAggAgACggTTCCgggggACC |
|  | 4 | ggCgTgggTCgACCTCCgCCTTgCCggTCgCTCgCCCTTTCCCCgggTCggg |
|  | 5 | ACCTAgCgCgTTCCggCgCggAggTTTAAAgACCCCTTggggggATCgCCCg |
|  | 6 | CgTggTgTgAAACCTTCCgACCCCTCTCCggAgTCCggTCCCgTTTgCTgTC |
|  | 7 | CgTCggCCCCggCCgggTggAAggTCCCgTgCCCgTCgTCgTCgTCgTCgTC |
|  | 8 | gCCCgTCgTgCTgCCCTCTCggggggTTTgCgCgAgCgTCggCTCCgCCTgg |
|  | 9 | CCCggAgCgggACCgggTCggAggATggACgAgAATCACgAgCgACggTggT |
|  | 10 | TgCgCCCgAgCgCggCCCggTggTCCCTgCCggACAggCgTTCgTgCgACgT |
|  | 11 | CggCCgCgTCggggCCTCgCCgCgCTCTACCTTACCTACCTggTTgATCCTg |
|  | 12 | CggCCgCgACAACCCCACCCCgCTggCTCCgTgCCgTgCgTgTCAggCgTTC |
|  | 13 | gTCTCCgCggggTTgTCCgCCgCCCCTTCCCCggAgTggggggTTggCCggA |
|  | 14 | CTTCACgTCCgTTggTggCCCCgCCTgggACCgAACCCggCACCgCCTCgTg |
|  | 15 | CCgTCCgTCCgTCCgCCgAgCggCCCgTCCCCCTCCgAgACgCgACCTCAgA |
|  | 16 | gTCCgCgCgTgggTCCTgAgggAgCTCgTCggTgTggggTTCgAggCggTTT |
|  | 17 | CCCgCCggCCgCgAgAgCCggAgAACTCgggAgggAgACgggggAgAgAgAg |
|  | 18 | gCgTCgTCggCggTgggggCgTgTTgCgTgCggTgTggTggTgggggAggAg |
|  | 19 | AgAAgCgAgggTTCCgCCggCCACCgCggTggTggCCgAgTgCggCTCgTCg |
|  | 20 | AgAgAgAgAAAgAgAAAgAAgggCgTgTCgTTggTgTgCgCgTgTCgTgggg |
